# A Truncated NLR Protein, TIR-NBS2, Is Required for Activated Defense Responses in the *exo70B1* Mutant

**DOI:** 10.1371/journal.pgen.1004945

**Published:** 2015-01-24

**Authors:** Ting Zhao, Lu Rui, Juan Li, Marc T. Nishimura, John P. Vogel, Na Liu, Simu Liu, Yaofei Zhao, Jeffery L. Dangl, Dingzhong Tang

**Affiliations:** 1 The State Key Laboratory of Plant Cell and Chromosome Engineering, Institute of Genetics and Developmental Biology, Chinese Academy of Sciences, Beijing, China; 2 Graduate School of Chinese Academy of Sciences, Beijing, China; 3 Howard Hughes Medical Institute and Department of Biology, University of North Carolina at Chapel Hill, North Carolina, United States of America; 4 Western Regional Research Center, United States Department of Agriculture, Agricultural Research Service, Albany, California, United States of America; University of British Columbia, CANADA

## Abstract

During exocytosis, the evolutionarily conserved exocyst complex tethers Golgi-derived vesicles to the target plasma membrane, a critical function for secretory pathways. Here we show that *exo70B1* loss-of-function mutants express activated defense responses upon infection and express enhanced resistance to fungal, oomycete and bacterial pathogens. In a screen for mutants that suppress *exo70B1* resistance, we identified nine alleles of *TIR-NBS2* (*TN2*), suggesting that loss-of-function of *EXO70B1* leads to activation of this nucleotide binding domain and leucine-rich repeat-containing (NLR)-like disease resistance protein. This NLR-like protein is atypical because it lacks the LRR domain common in typical NLR receptors. In addition, we show that TN2 interacts with EXO70B1 in yeast and *in planta*. Our study thus provides a link between the exocyst complex and the function of a ‘TIR-NBS only’ immune receptor like protein. Our data are consistent with a speculative model wherein pathogen effectors could evolve to target EXO70B1 to manipulate plant secretion machinery. TN2 could monitor EXO70B1 integrity as part of an immune receptor complex.

## Author Summary

Secretory pathways play an important role in the plant immune response by delivering antimicrobial compounds and metabolites to the site of infection. The evolutionarily conserved exocyst complex is involved in exocytosis, the final step in the secretory pathway. We showed that loss of the function of EXO70B1, a subunit of exocyst complex, results in activated defense responses, and enhanced resistance to a range of pathogens. We found that EXO70B1 associates with the SNARE complex protein SNAP33, which is involved in focal secretion of defense-related proteins. Enhanced disease resistance and cell death in the *exo70B1* mutant are dependent on TIR-NBS2 (TN2), an atypical intracellular immune receptor-like protein that lacks leucine-rich repeats. TN2 physically associates with EXO70B1, and *TN2* transcripts accumulate at much higher levels in the *exo70B1* mutant. These data are consistent with a model where activation of a receptor pathway containing TIR-NBS2 is responsible for activated defense responses and cell death in *exo70B1*. Our data further suggest that this, and possibly other, exocyst components could be targets of effectors that are guarded by immune receptors.

## Introduction

Powdery mildew fungi are obligate biotrophic pathogens that cause widespread disease in many plant species, including economically important crops such as barley [1], wheat [2] and tomato [3] as well as model plants such as *Arabidopsis* [4]. Powdery mildew fungal spores germinate on the leaf surface, and then extend hyphae, which form appressoria at the attempted penetration sites. These appressoria penetrate the epidermal cell wall. The fungus then invaginates the plant plasma membrane and develops a haustorium, a feeding structure [5]. Evolutionarily adapted powdery mildew fungal pathogens can complete the infection cycle by producing conidia, asexual spores, on the host leaf surfaces. For instance, the adapted powdery mildew pathogen for *Arabidopsis, Golovinomyces cichoracearum*, produces abundant conidia on Col-0 leaves after infection. Several types of barley and *Arabidopsis* mutants show altered responses to adapted powdery mildew pathogens; these include *mlo* (mildew locus O) [1,6], *pmr* (powdery mildew resistant) [6–10] and *edr* (enhanced disease resistance) [11–14]. These mutants generally are more resistant to adapted powdery mildew pathogens, indicating that *MLO, PMR*, and *EDR* have negative roles in powdery mildew resistance.

Adapted powdery mildew fungi can infect the host plant; by contrast, non-adapted powdery mildew pathogens usually cannot penetrate plant epidermal cell walls or form haustoria. Genetic analyses demonstrated that the full penetration resistance of *Arabidopsis* to non-adapted powdery mildew requires the functions of PEN1, PEN2 and PEN3 [15–17]. *PEN1* encodes syntaxin SYP121, which participates in vesicle fusion events by forming ternary soluble *N*-ethylmaleimide–sensitive factor attachment protein receptor (SNARE) complexes with the synaptosome-associated membrane protein 33 (SNAP33) and vesicle-associated membrane protein721 (VAMP721) and VAMP722 [15,18].

Before fusion occurs, SNARE-mediated vesicles must dock at the plasma membrane; this requires the exocyst, which mediates the early steps of exocytosis [19]. The exocyst complex, originally defined in yeast [20], consists of 8 proteins: Sec3, Sec5, Sec6, Sec8, Sec10, Sec15, EXO70, and EXO84. Among these eight proteins, Sec3 and EXO70 are targeted to the plasma membrane by binding to phosphatidyl (4,5) biphosphate [21,22].

Homologs of all eight exocyst proteins have been found in plants [23–25]. Plant exocyst proteins function in regulation of polarity and morphogenesis [26]. In *Arabidopsis*, Sec6, Sec8, and EXO70A1 localize to the tips of growing pollen tubes and function in polar secretion required for growth and pollen incompatibility [25,27]. Sec3 and EXO70A1 function in polarized secretion in elongating root hairs [28] and in the interaction between the stigma and compatible pollen [29]. Sec6, Sec8, Sec15b, EXO70A1, and EXO84b also function in secretory processes during cytokinesis, and Sec8 and EXO70A1 are required for the localized deposition of seed coat pectin [30,31]. In addition, EXO70A1 also affects auxin efflux carrier recycling, polar auxin transport [32] and tracheary element development [33].

EXO70 clade members also function in plant immunity [26]. For instance, *Arabidopsis EXO70B2* and *EXO70H1* are transcriptionally induced by *Pseudomonas syringae pv. maculicola* and the fungal pathogen *Bgh* and are required for full plant defense responses. Further, EXO70B2 associates with SNAP33, and contributes to plant immunity [34]. EXO70B2 is a target of the plant U-box-type ubiquitin ligase 22 (PUB22) and is required for PAMP-triggered responses [34,35]. EXO70B1 also affects autophagy-related membrane traffic to the vacuole, and the *exo70B1* mutant displays ectopic hypersensitive reaction [36].

Plant disease resistance (R) proteins play critical roles in plant immunity [37]. The R proteins directly or indirectly recognizes pathogen effectors, and leads to effector triggered immunity. Most of the R proteins are intracellular, and belongs to nucleotide binding (NB) domain and leucine-rich repeat (LRR)-containing (NLR) immune receptors, which can be divided to two subclasses, based on the N-terminal domains. The first class of NLR immune receptors contains a Toll-Interleukin-1 Receptor (TIR) domain and the second class contains a coiled-coil (CC) domain [37]. Activation of NLR leads to hypersensitive responses which is characterized by localized cell death at site of infection [37]. Besides those full length NLR immune receptors, there are a number of atypical NLRs that lack LRR domain in plant genome [38]. Although those truncated NLR proteins are implicated in plant immunity, the function of those proteins are not well understood [39].

Here, we report that the *exo70B1* mutant displays enhanced resistance to powdery mildew. During the course of this work, it was shown that *exo70B1* displayed spontaneous cell death, which was delayed by *npr1* mutation suggesting that at least part of the mutant phenotype is due to constitutive SA signaling [36]. Consistent with this, *exo70B1* exhibits up-regulation of *PR1*, and accumulates higher levels of SA in the absence of pathogen attack [36]. Here, we show that EXO70B1 associates with the SNARE complex protein SNAP33. In a suppressor screen, we found that the *exo70B1*-associated phenotypes require the TIR-NBS gene *TN2*. These data indicate that mutation of EXO70B1 leads to activation of a defense pathway that requires TN2, a truncated form of the classical TIR-NBS-LRR intracellular immune system receptors.

## Results

### The *exo70B1-3* mutant displays enhanced disease resistance to *Golovinomyces cichoracearum*

**Figure 1:**
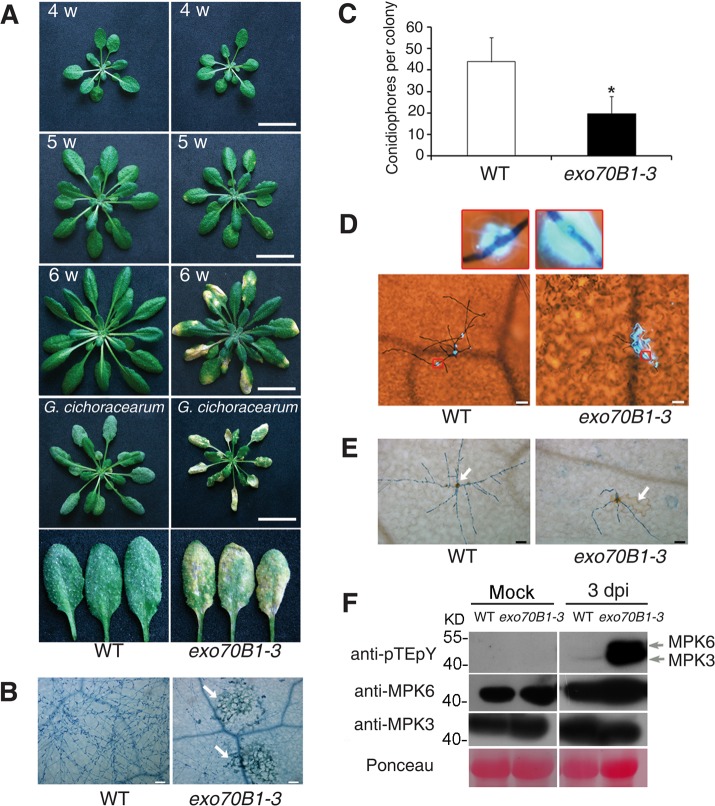
*exo70B1-3* mutants display enhanced resistance to *G. cichoracearum*. **(A)** Plants were grown in the standard short day conditions. Upper three panels: Uninfected four-, five- and six-week-old wild type and *exo70B1-3* plants were photographed. *exo70B1-3* mutants started to develop spontaneous cell death at five weeks of age, and cell death was more pronounced at six weeks of age. Lower two panels: Four-week-old plants were infected with *G. cichoracearum* and the plants or representative leaves were photographed at 7 dpi. The wild type plants displayed a large number of fungal spores on the leaves, but the *exo70B1-3* mutants displayed very few spores, with massive necrotic lesions on the leaves. Five-week-old wild type and *exo70B1-3* plants were the uninoculated controls for plants infected with *G. cichoracearum*. Bar = 20 mm. **(B)** Infected leaves at 7 dpi were stained with trypan blue to show the fungal structures and dead cells (arrows). Bar = 100 μm. **(C)** Fungal growth in plants was assessed by counting the number of conidiophores per colony at 5 dpi. The asterisk indicates a significant difference from wild type (*p <* 0.01; Student’s *t*-test). Bars represent mean and standard deviation (n>30). The experiments were repeated three times with similar results. **(D)** Infected leaves were stained with aniline blue at 2 dpi to examine callose deposition (blue dots, indicated by red quadrangle). Bar = 50 μm. **(E)** Infected leaves were stained with 3,3′-diamino benzidine-HCl (DAB) at 2 dpi to visualize accumulation of hydrogen peroxide (brown staining, indicated by arrow). Bar = 50 μm. **(F)** The *exo70B1-3* mutants showed enhanced MAP kinase activation upon *G. cichoracearum* infection. Four-week-old plants were infected with *G. cichoracearum* at high inoculum densities. The immunoblot analysis was performed using anti-pTEpY antibody to examine MAPK activation, and anti-MPK3 or anti-MPK6 to show accumulation of MPK3 or MPK6 protein, respectively. Individual MPKs are indicated by arrows. Ponceau S staining of RuBisCO is shown as loading control.

To study the molecular interactions between *Arabidopsis* and powdery mildew fungus, we screened for mutants that displayed enhanced disease resistance and an exaggerated cell death response following infection with *G. cichoracearum* UCSC1 [14]. We designated one of the recessive mutants identified in this screen *exo70B1-3*, based on our subsequent characterization. Under standard short day conditions, *exo70B1-3* grew normally up to 4 weeks of age. After 4 weeks, *exo70B1-3* plants were slightly smaller than the wild type, and displayed HR-like cell death after 5 weeks (Fig. 1A and S1 Fig.). After inoculation with *G. cichoracearum* at high inoculum density, the leaf surface of the wild type was covered with abundant fungal spores at 7 dpi, but *exo70B1-3* exhibited extensive necrotic lesions and no visible powder of fungal spores on leaves (Fig. 1A). Trypan blue staining revealed abundant fungal hyphae and conidiophores on the leaves of the wild type, but showed very few fungal hyphae and large patches of dead cells on the leaves of *exo70B1-3* (Fig. 1B). To further assess the powdery mildew resistance of *exo70B1-3*, we quantified fungal growth in plants at 5 dpi. Consistent with the trypan blue staining, *exo70B1-3* supported significantly fewer conidiophores than the wild type (Fig. 1C), indicating that fungal reproduction was inhibited in the *exo70B1-3* mutant.

Plant responses to pathogen infection include: callose deposition, hydrogen peroxide (H_2_O_2_) accumulation, and MAPK activation [40]. We used aniline blue to visualize callose deposition in the infected leaves of *exo70B1-3* at 2 dpi. As shown in Fig. 1D, very little callose was deposited at the infection sites of pathogen in the wild type, but a large amount of callose was produced at the sites of fungal infection in *exo70B1-3* at 2 dpi, when cell death did not occur. In plants infected with pathogens, cell death triggered by specific recognition is usually associated with H_2_O_2_ accumulation [41,42]. We examined H_2_O_2_ accumulation in wild type and *exo70B1-3* by staining the infected leaves with 3,3’-diamino benzidine hydrochloride (DAB) and trypan blue and found that *exo70B1-3* leaves accumulated more H_2_O_2_ upon fungal attack than wild type (Fig. 1E).

We also examined the activation of MPKs upon infection with *G. cichoracearum*, by immunoblotting with anti-pTEpY antibody, which detects phosphorylated MPK3 and MPK6 [43]. As shown in Fig. 1F, MPK3 and MPK6 were activated in both wild type and *exo70B1-3* at 3 dpi; however, activation of MPK3, MPK6 in *exo70B1-3* was much stronger than in wild type. Protein levels of MPK3 and MPK6 were similar in infected and uninfected wild type and mutant plants (Fig. 1F).

**Figure 2:**
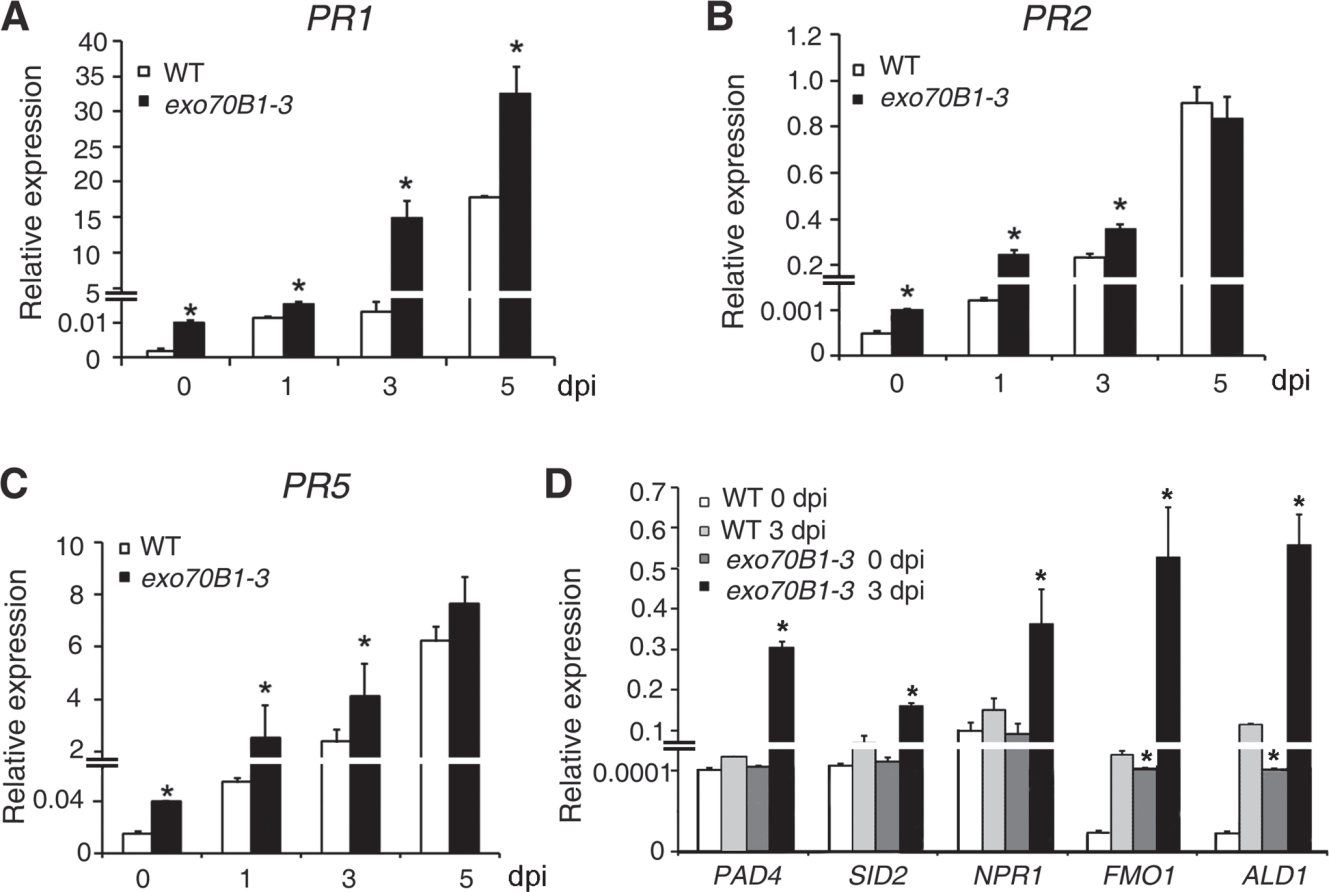
Expression of defense-related genes in wild type and *exo70B1-3* mutants. **(A)-(D)** The transcript accumulation of defense-related genes was examined by quantitative real-time RT-PCR. Leaves were detached from four-week-old plants for RNA isolation at different time points (day 0: uninfected) after infection with *G. cichoracearum. PR1* **(A)**, *PR2* **(B)**, *PR5* **(C)**, *PAD4, SID2, NPR1, FMO1, ALD1* **(D)**. *ACT2* was used as an internal control. Bars represent mean and standard deviation of values obtained from three independent biological samples. Three technical replicates for each biological sample were examined in the experiment. The asterisks indicate a significant difference from WT (*p <* 0.01; Student’s *t*-test). The experiments were repeated three times with similar results.

To examine whether *exo70B1-3* affects the expression of immune output genes, we measured the transcript levels of the pathogenesis-related gene *PR1*, a marker for salicylic acid signaling, at various time points from 0 to 5 dpi with *G. cichoracearum*, using quantitative real time RT-PCR. Prior to inoculation, *PR1* transcript levels were very low in both genotypes, but the basal expression levels of *PR1* were higher in *exo70B1-3* than in wild type. Upon infection with *G. cichoracearum, PR1* transcript accumulated at much higher levels in *exo70B1-3* than in wild type (Fig. 2A). We also examined expression of other immune output genes, including *PR2* (Fig. 2B), *PR5* (Fig. 2C), *ALD1, FMO1, PAD4* and *SID2* (Fig. 2D). In general, the relative transcript levels of those genes were low at day 0, but were much higher in *exo70B1-3* than in wild type at day 0 and 3 dpi. However, for *PAD4, SID2* and *NPR1*, we observed significant differences in transcript levels between *exo70B1-3* and wild type only at day 3, not at day 0 (Fig. 2D). Taken together, these data indicated that *exo70B1-3* displayed enhanced disease resistance, and upon infection, defense responses were activated at higher levels in *exo70B1-3* than in wild type.

### PAD4, EDS5 and NPR1 contribute to *exo70B1-3* mediated powdery mildew resistance, but only PAD4 is required for ectopic cell death

**Figure 3:**
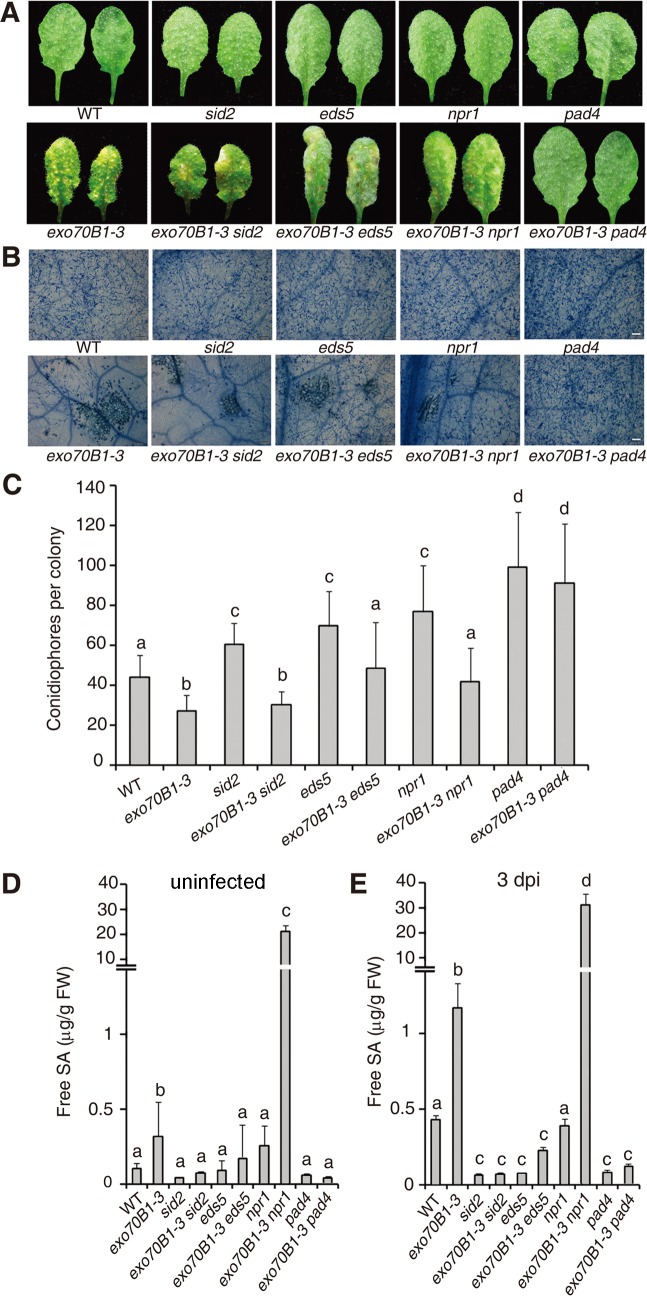
The *exo70B1-3* mutants accumulate high levels of SA and their enhanced resistance to *G. cichoracearum* requires *EDS5, NPR1* and *PAD4*. **(A)** Four-week-old plants were infected with *G. cichoracearum* at high inoculum densities, and representative leaves removed from plants at 7 dpi with *G. cichoracearum*. **(B)** Leaves removed from plants infected with *G. cichoracearum* at high inoculum densities at 7 dpi were stained with trypan blue to show hyphae and dead cells. Bar = 100 μm. **(C)** Fungal growth in plants infected with *G. cichoracearum* at low inoculum densities at 5 dpi was assessed by counting the number of conidiophores per colony. Lower-case letters indicate statistically significant differences (*p <* 0.05; one-way ANOVA). **(D)-(E)** *exo70B1-3* mutants accumulate high levels of free SA. SA was extracted from leaves of uninfected four-week-old plants **(D)**, or leaves of four-week-old plants infected with *G. cichoracearum* at high inoculum densities at 3 dpi **(E)**. Bars represent mean and standard deviation from three biological replicates for each genotype. Lower-case letters indicate statistically significant differences (*p* < 0.05; one-way ANOVA). These experiments were repeated three times with similar results.

Salicylic acid (SA) plays critical roles in the defense response to pathogens and activation of SA signaling pathways often leads to disease resistance and cell death in response to pathogen attack [44]. To assess the role of SA signaling in the disease resistance observed in *exo70B1-3*, we crossed *exo70B1-3* with *pad4, sid2* and *eds5* mutants, which express lower levels of pathogen-induced SA [45–47] or *npr1*, which has defects in SA signaling [48]. Double mutants were then infected with *G. cichoracearum* and phenotypes were scored at 7 dpi. Only *pad4* completely suppressed both powdery mildew resistance and mildew-induced cell death in *exo70B1-3* (Fig. 3A-C). The *eds5* and *npr1* mutations suppressed powdery mildew resistance, but did not fully suppress cell death in *exo70B1-3*. The *sid2* mutation affected neither resistance nor cell death in *exo70B1-3* (Fig. 3A-C). Similarly, spontaneous cell death in *exo70B1-3* was only suppressed by *pad4*, but not by *sid2, npr1* or *eds5* (S2 Fig.). Taken together, these observations indicate that PAD4, EDS5 and NPR1 contribute to *exo70B1-3* mediated powdery mildew resistance. Those observations also suggest that the cell death and ectopic disease resistance in *exo70B1-3* can be uncoupled.

To further assess the role of SA in *exo70B1-3* mediated resistance, we measured SA levels in *exo70B1-3*. Free SA levels were significantly higher in *exo70B1-3* plants than in wild type in the absence of pathogen or at 3 dpi with *G. cichoracearum* (Fig. 3D, 3E). The SA levels in *exo70B1-3* were reduced to wild type levels (or even lower) in combination with *pad4, eds5* and *sid2*. However, the SA levels in *exo70B1-3 npr1* were much higher than in *exo70B1-3*, which was consistent with previous findings that *npr1* accumulates higher levels of SA, and that *npr1* mutation often further increases SA levels in mutants that over-accumulate SA [49–51]. The observation that *exo70B1-3 sid2* mutants accumulated low levels of SA, but still displayed enhanced disease resistance and mildew-induced cell death, indicates that the disease resistance and cell death phenotypes in response to *G. cichoracearum* in *exo70B1-3*, similar to the *lsd1*-mediated immune responses, do not result from high accumulation of SA [52].

We examined the role of ethylene and JA in *exo70B1-3* mediated defenses by examining the effect of mutations in *EIN2* and *COI1*, which block all known ethylene and JA responses, respectively [53,54]. The *exo70B1-3 coi1* and *exo70B1-3 ein2* double mutants did not show any alteration in disease resistance or cell death in response to *G. cichoracearum* infection compared to *exo70B1-3* (S3 Fig.), indicating that the ethylene and JA pathways do not affect cell death and disease resistance observed in *exo70B1-3*.

We also crossed *exo70B1-3* with two well-characterized powdery mildew resistant mutants, *edr1* and *pmr4*, and then infected the double mutants with *G. cichoracearum*. As shown in S4 Fig., the *edr1* and *pmr4* mutations enhanced *exo70B1-3* resistance phenotypes, indicating that the disease resistance in *exo70B1-3* may differ from the previously characterized powdery mildew resistance mediated by *edr1* and *pmr4*.

### *exo70B1-3* displayed enhanced disease resistance to the oomycete pathogen *H. arabidopsidis* Noco2 and the bacterial pathogen *Pseudomonas syringae* pv. *tomato strain* DC3000

**Figure 4:**
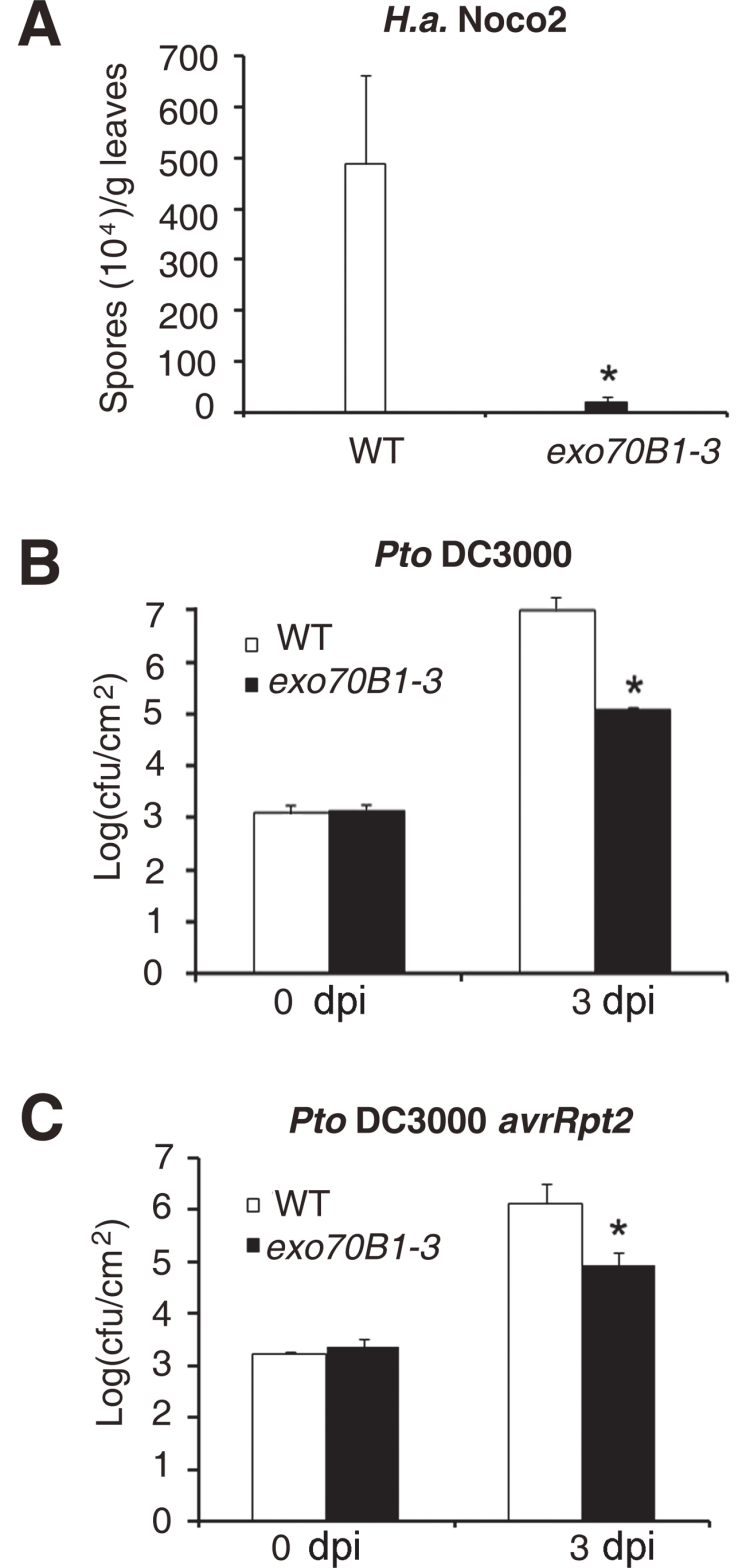
*exo70B1-3* mutants display enhanced resistance to *H.a.* Noco2, *Pto* DC3000 and *Pto* DC3000 *avrRpt2*. **(A)** Two-week-old seedlings were inoculated with *H.a.* Noco2. The number of spores was counted at 7 dpi. The asterisk indicates a significant difference from WT (*p <* 0.01; Student’s *t*-test). **(B)-(C)** Four-week-old plants were infiltrated with *Pto* DC3000 **(B)** and *Pto* DC3000 *avrRpt2* **(C)**. Bacterial growth was monitored at 0 and 3 dpi. cfu: colony-forming units. The asterisk indicates a significant difference from wild type (*p <* 0.01; Student’s *t*-test). Bars represent mean and standard deviation of values from three biological samples. The experiments were repeated three times with similar results.

To examine whether *exo70B1-3* causes resistance to other pathogens, we challenged *exo70B1-3* with the virulent oomycete pathogen *Hyaloperonospora arabidopsidis* (*H. a.*) Noco2 and the bacterial pathogen *Pseudomonas syringae* pv. *tomato* (*Pto*) DC3000. Two-week old seedlings were infected with *H.a.* Noco2, and we found that *exo70B1-3* supported significantly fewer spores than the wild type at 7 dpi (Fig. 4A). Similarly, *exo70B1-3* were more resistant to the virulent strain *Pto* DC3000 and the avirulent bacterial strain *Pto* DC3000 *avrRpt2* (Fig. 4B, 4C).

### EXO70B1 is an exocyst subunit

We identified the *exo70B1-3* mutation by map-based cloning (S5A Fig.). To confirm that the *EXO70B1* we defined is the gene responsible for the *exo70B1-3* phenotype, we examined the phenotypes of two additional *EXO70B1* T-DNA insertion lines N328818 (*exo70B1-1*) and N717829 (*exo70B1-2*) (S5A-S5B Fig.), and noted that these two mutants also displayed enhanced disease resistance and lesions upon inoculation with *G. cichoracearum*, a phenotype similar to that of *exo70B1-3* (S5C Fig.). As an additional confirmation, we introduced an *EXO70B1* genomic clone into *exo70B1-3* plants, and found that the *EXO70B1* genomic clone complemented the *exo70B1-3* phenotypes (S5C Fig.). We also examined *PR1* expression in the three *exo70B1* alleles, the *exo70B1-3 gEXO70B1* transgenic line and wild type upon mildew infection. We found that accumulation of the *PR1* transcripts in the *exo70B1-3* transgenic lines was similar to that in the wild type, which was much lower compared to the three *exo70B1* alleles (S5D Fig.). Taken together, these data demonstrated that loss of *EXO70B1* is responsible for the enhanced resistance to powdery mildew in *exo70B1-3* mutants.

The 23 annotated *Arabidopsis EXO70* genes fall into nine clusters, and *EXO70B1* falls into the *EXO70B* cluster, which is very conserved in land plants [55]. Among the 23 EXO70 family members in *Arabidopsis*, EXO70B2 shares the highest sequence similarity with EXO70B1. However, *exo70B2-1* was as susceptible as wild type in response to *G. cichoracearum*, supporting abundant fungal growth on the leaf surface and developing no visible lesions at 7 dpi. The *exo70B1-3 exo70B2-1* double mutant was similar to *exo70B1-3* (S6A-S6C Fig.).

As EXO70B1 is predicted to be a subunit of the exocyst complex, we performed yeast two-hybrid assays to examine whether EXO70B1 interacts with other exocyst subunits. Although EXO70B1 seems to be weakly auto-activating in our experimental conditions, the yeast two-hybrid assays showed that EXO70B1 interacts with SEC3a, SEC5a, SEC15b and EXO84bN (S7 Fig.). However, Kulich et al. previously showed that EXO70B1 interacted with only SEC5a and EXO84bN, but not SEC3a. [36]. The discrepancy may be because the interaction between EXO70B1 and SEC3a is relatively weak.

To further examine *EXO70B1* expression, we expressed a GUS reporter gene in wild type under the control of the *EXO70B1* promoter. We obtained a number of *EXO70B1*::GUS transformants and analyzed 20 transgenic lines. We observed GUS activity in seedlings, leaves, calyx, stigma and siliques, demonstrating that *EXO70B1* is ubiquitously expressed (S8 Fig.).

### EXO70B1 interacts with SNAP33

**Figure 5:**
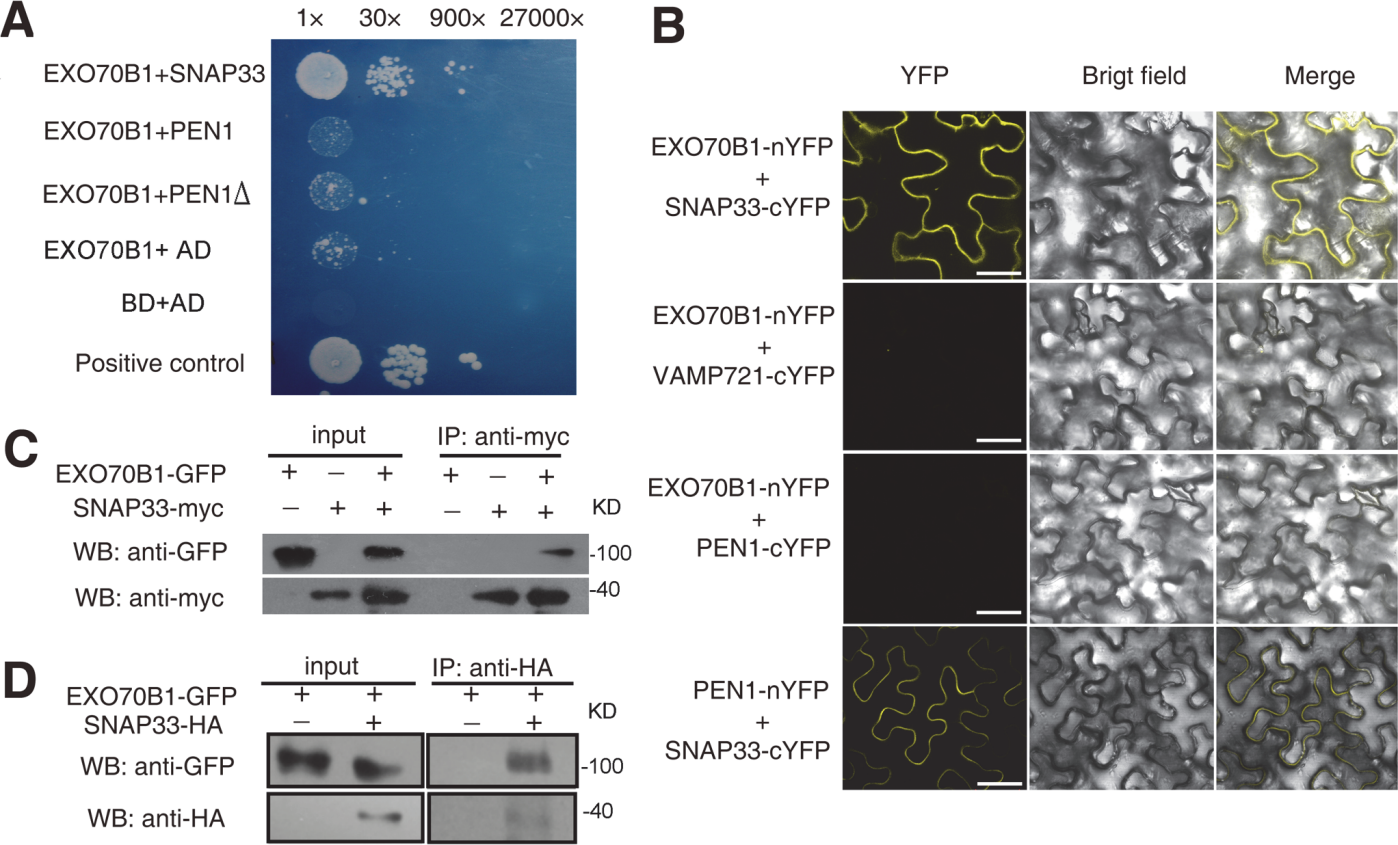
EXO70B1 associates with SNAP33. **(A)** EXO70B1 interacted with SNAP33 in yeast two-hybrid assays. Overnight culture from a single colony was diluted with sterile water to OD = 0.5, and serial dilutions 1:30, 1:900, 1:27,000 were prepared and dropped onto SD-Ade-His-Leu-Trp plates at 28°C, respectively. Each drop was 10 μL. The photograph was taken at day 5 after plating. PEN1Δ: PEN1 without transmembrane domain. **(B)** EXO70B1 and SNAP33 interaction was examined with BiFC assays in *N. benthamiana*. EXO70B1 was fused to the N-terminal fragment of YFP (nYFP); SNAP33, PEN1 and VAMP721 were fused to the C-terminal fragment of YFP (cYFP). Cauliflower mosaic virus 35S promoter (35Spro) was used in this assay. YFP fluorescence was observed only with the transiently expressed EXO70B1-nYFP and SNAP33-cYFP, but not EXO70B1-nYFP and VAMP721-cYFP nor EXO70B1-nYFP and PEN1-cYFP in *N. benthamiana*. The combination of PEN1-nYFP and SNAP33-cYFP was used as the positive control. Bar = 50 μm. **(C)** EXO70B1 interacted with SNAP33 in a Co-IP assay in *N. benthamiana. EXO70B1pro:EXO70B1-GFP* was co-expressed with *35Spro:SNAP33-Myc* in *N. benthamiana* leaves. Total protein was extracted and subjected to immunoprecipitation of SNAP33 protein by anti-Myc antibody. Proteins were analyzed in an immunoblot using antibodies as indicated. **(D)** Co-IP of EXO70B1 and SNAP33 using transgenic *Arabidopsis* plants. The *exo70B1-3* transgenic plants expressing *EXO70B1pro:EXO70B1-GFP* and *SNAP33pro:SNAP33-HA* were used in the Co-IP assay. Total protein was extracted from 3-week-old transgenic plants that express both EXO70B1-GFP and SNAP33-HA, or EXO70B1-GFP alone (negative control). The SNAP33 protein was immunoprecipitated by anti-HA antibody, followed by immunoblot analysis with the GFP antibody to detect the presence of EXO70B1-GFP in the precipitate.

The ternary SNARE complex PEN1-SNAP33-VAMP721/VAMP722 affects powdery mildew resistance [56]. To examine whether EXO70B1 functions in the PEN1-SNAP33-VAMP721/VAMP722 pathway, we first used yeast two-hybrid assays to test whether EXO70B1 interacts with the PEN1, SNAP33 and VAMP721. As shown in Fig. 5A, EXO70B1 interacted with SNAP33, but not PEN1 or PEN1 without its transmembrane domain. To confirm the interaction between EXO70B1 and SNAP33, we used bimolecular fluorescence complementation (BiFC) in *Nicotiana benthamiana* [57] to examine whether EXO70B1 and SNAP33 can interact. EXO70B1 and SNAP33 were fused to the N-terminal and C-terminal fragments of YFP, respectively. In *N. benthamiana* leaves co-transformed with the *EXO70B1-YFP^N^* and *SNAP33-YFP^C^* or *PEN1-YFP^N^* and *SNAP33-YFP^C^* constructs, we observed YFP fluorescence (Fig. 5B and S9 Fig.). However, we observed no YFP fluorescence in leaves co-transformed with *EXO70B1-YFP^N^* and *VAMP721-YFP^C^* or *PEN1-YFP^C^*, suggesting that EXO70B1 interacts with SNAP33 but not PEN1 in *N. benthamiana*. These results were consistent with the observations from the yeast two-hybrid assays.

To confirm the interaction of EXO70B1 and SNAP33, we also performed co-immunoprecipitation (Co-IP) in *N. benthamiana*. We co-expressed EXO70B1-GFP with SNAP33-Myc in *N. benthamiana* leaves and expressed EXO70B1-GFP or SNAP33-Myc alone as negative controls. SNAP33 protein was immunoprecipitated by anti-Myc antibody, and anti-GFP antibody detected EXO70B1-GFP in the precipitate only from the leaves that expressed both EXO70B1-GFP and SNAP33-Myc, not from the negative controls (Fig. 5C), indicating that EXO70B1 and SNAP33 associate in *N. benthamiana*. We also examined whether EXO70B1 interacts with PEN1 by Co-IP assays; however, as shown in S10 Fig., EXO70B1 did not co-immunoprecipitate with PEN1, indicating that EXO70B1 and PEN1 did not associate, consistent with the results from the yeast two-hybrid and BiFC assays.

To further confirm the association of EXO70B1 and SNAP33, we performed Co-IP assays using stably transformed transgenic *Arabidopsis* plants expressing both EXO70B1-GFP and SNAP33-HA under their respective native promoters. The EXO70B1-GFP construct complemented the *exo70B1-3* mutant phenotypes (S11 Fig.), indicating that the EXO70B1-GFP protein is functional. Total protein was extracted from the transgenic plants and SNAP33-HA protein was immunoprecipitated by anti-HA antibody. We then examined whether EXO70B1-GFP was in the precipitate by immunoblot analysis with the GFP antibody. EXO70B1-GFP protein was only detected in the transgenic plants that expressed both EXO70B1-GFP and SNAP33-HA, not in the negative control (Fig. 5D), indicating that EXO70B1-GFP associates with SNAP33-HA in *Arabidopsis*.

**Figure 6:**
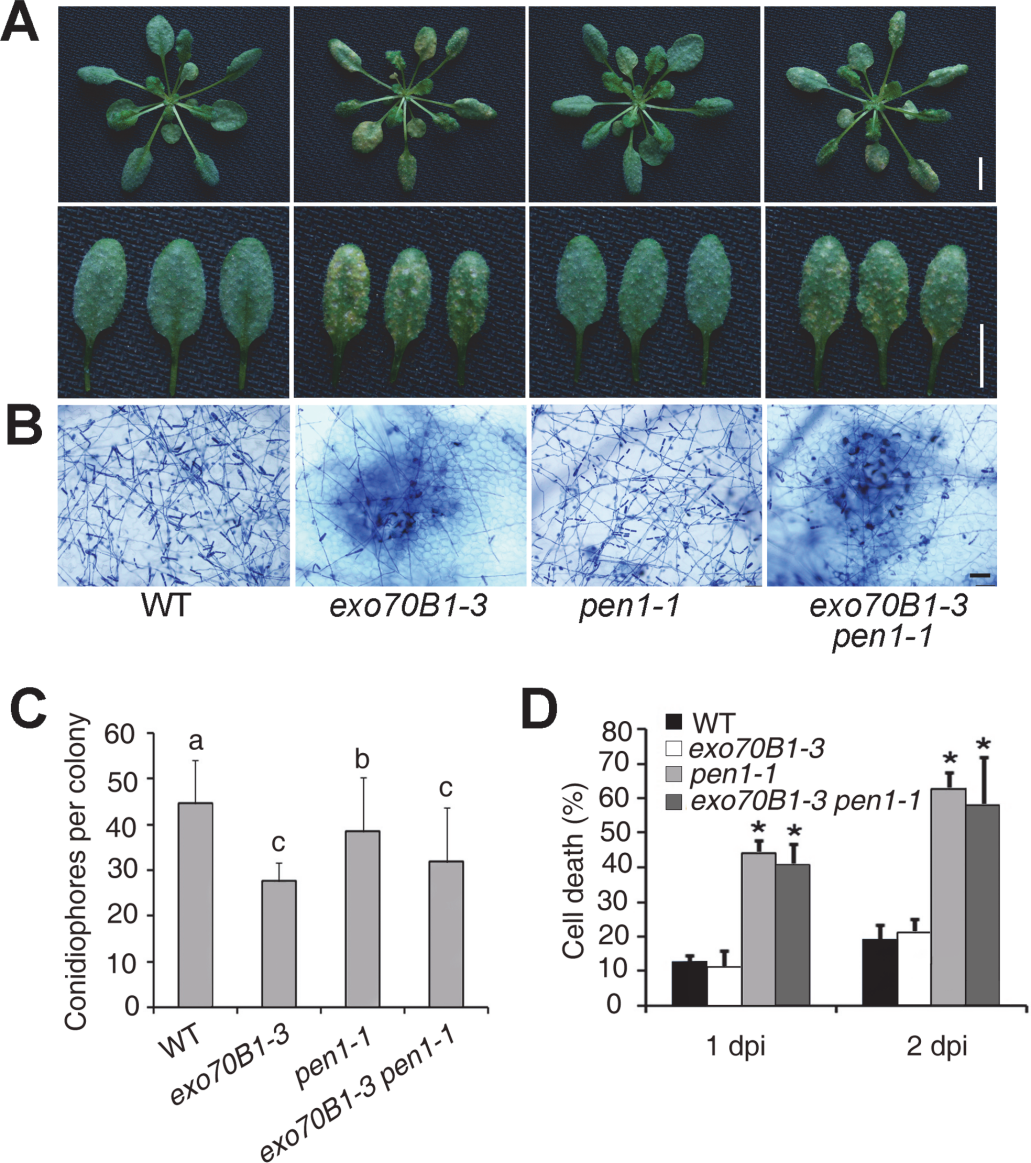
Defense responses of wild type, *exo70B1-3, pen1-1* and *pen1-1 exo70B1-3* plants to adapted and non-adapted powdery mildew pathogens. **(A)-(C)** Four-week-old plants were infected with *G. cichoracearum*. The *pen1-1* mutation did not affect powdery mildew resistance in *exo70B1-3*. **(A)** Leaves at 7 dpi were removed and photographed. Bar = 10 mm. **(B)** Plant cell death and fungal growth were examined with trypan blue staining. Bar = 50 μm. **(C)** Fungal growth in plants was quantified by counting the number of conidiophores per colony at 5 dpi. Bars represent mean and sd (n = 30). Statistically significant difference from wild type is indicated by lower case letters (*p* < 0.05; one-way ANOVA). The experiments were repeated three times with similar results. **(D)** Four-week-old plants were infected with *Blumeria graminis f. sp. tritici*. Frequency of epidermal single cell death induced by infection with *Blumeria graminis f. sp. Tritici* was calculated at 1 and 2 dpi. The *pen1-1* mutant showed a significantly higher frequency of epidermal single cell death upon *Blumeria graminis f. sp. Tritici* infection; by contrast, the frequency of epidermal single cell death in *exo70B1-3* was similar to that of wild type. Bars represent mean and standard deviation (n = 6, scoring 40 sites per time point). Asterisks indicate statistically significant difference from wild type (*p* < 0.01; Student’s *t*-test). The experiments were repeated three times with similar results.

EXO70B1 interacts with SNAP33, a component of the PEN1-containing ternary SNARE complex involved in penetration resistance. To examine whether the *exo70B1* mutant phenotype is altered in the absence of PEN1, we made *pen1-1 exo70B1-3* double mutants, and then infected wild type, *exo70B1-3, pen1-1*, and *pen1-1 exo70B1-3* with adapted and non-adapted powdery mildew pathogens. Both *exo70B1-3* and *pen1-1* displayed enhanced resistance to *G. cichoracearum*, and *pen1-1 exo70B1-3* was very similar to *pen1-1* or *exo70B1-3* (Fig. 6A-C). However, *exo70B1-3* mutants were similar to wild type when infected with non-adapted wheat powdery mildew pathogen *Blumeria graminis f. sp. tritici*. But *pen1-1* showed a significantly higher frequency of epidermal single cell death, and *exo70B1-3 pen1-1* was very similar to *pen1-1* (Fig. 6D), suggesting that the loss of EXO70B1 may not affect PEN1-mediated resistance to a non-adapted pathogen, which is consistent with the observations that EXO70B1 does not interact with PEN1.

### Enhanced disease resistance in *exo70B1* requires the *TIR-NBS* gene *TN2*

**Figure 7:**
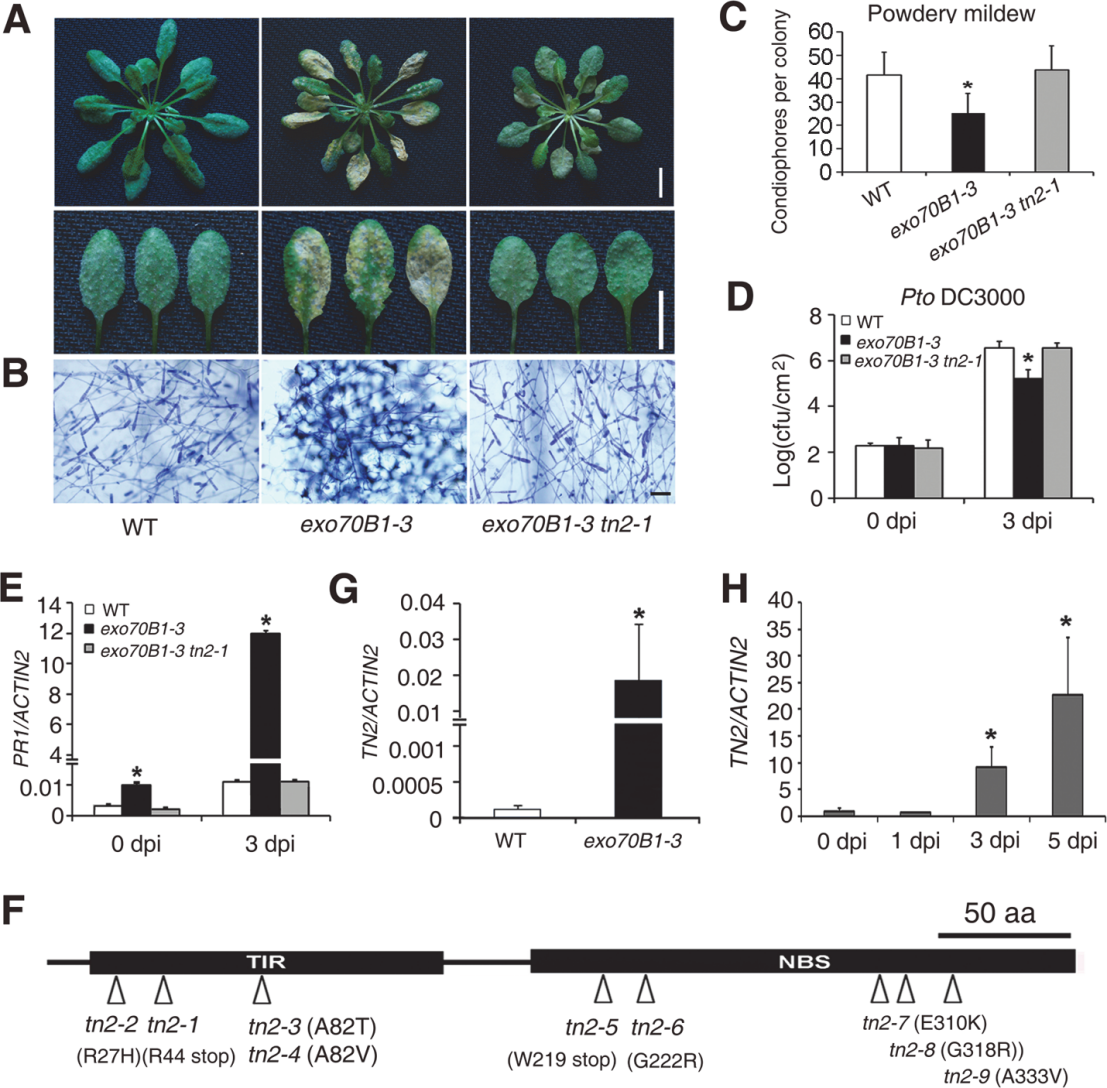
*exo70B1*-mediated resistance and cell death require the TIR-NBS protein TN2. **(A-C)** Four-week-old plants were infected with *G. cichoracearum*.**(A)** Intact plant (upper panel) or representative leaves (lower panel) from the infected plants were photographed at 7 dpi. The *exo70B1-3* mutant was resistant, but the *exo70B1-3 tn2-1* mutant was susceptible to *G. cichoracearum*. Bar = 10 mm. **(B)** Leaves from infected plants were stained with trypan blue at 7 dpi. Many fungal spores were produced in wild type and *exo70B1-3 tn2-1*, but few spores were produced in *exo70B1-3*. Bar = 50 μm. **(C)** Fungal growth was assessed by counting the number of conidiophores per colony at 5 dpi. Bars represent mean and standard deviation from three independent biological replicates (n = 30). The asterisk indicates a statistically significant difference (*p* < 0.05; Student’s *t*-test). **(D)** Four-week-old plants of wild type, *exo70B1-3* and *exo70B1-3 tn2-1* were inoculated with *Pto* DC3000 at OD_600_ = 0.0005. Bacterial growth was assessed at days 0 and 3. Bars represent means and standard deviation of three independent biological replicates. The asterisk indicates a statistically significant difference (*p* < 0.05, Student’s *t*-test). **(E)** Quantitative RT-PCR analysis of *PR1* expression in the infected plants with *G. cichoracearum*. The relative transcript levels were examined by real-time PCR and normalized to *ACTIN2*. Bars represent mean and standard deviation from three independent experiments. The asterisk indicates statistically significant difference (*p* < 0.05, Student’s *t*-test). **(F)** Protein structure of TN2. An arrowhead indicates the position of the mutated amino acid in each *tn2* mutant. **(G)-(H)** The transcript levels of *TN2* were examined by quantitative real-time PCR, and normalized to *ACTIN2*. Uninfected wild type and *exo70B1-3* plants (G) and wild type plants infected with *G. cichoracearum* (day 0: uninfected control) (H). Bars represent mean and standard deviation from three biological experiments. The asterisk indicates a statistically significant difference (*p* < 0.05, Student’s *t*-test).

We performed a genetic screen to identify suppressors of *exo70B1*. We mutagenized *exo70B1-3* seeds with EMS, and screened the M2 plants for mutants that displayed wild-type like phenotypes in response to *G. cichoracearum*. We obtained more than 20 mutants, which fell into 5 complementation groups, including nine alleles of At1g17615, which we defined by standard map-based cloning (S12 Fig.), seven alleles of *PAD4* (consistent with our double mutant analysis, above) and four mutant alleles of a gene encoding a calcium dependent protein kinase (which will be reported elsewhere). The mutation in At1g17615 fully suppresses *exo70B1*-associated phenotypes, including spontaneous cell death (S13A Fig.), powdery mildew resistance and cell death (Fig. 7A-C), increased accumulation of H_2_O_2_ and SA (S13B-S13C Fig.) and enhanced resistance to *Pto* DC3000 (Fig. 7D), indicating that enhanced disease resistance in *exo70B1* requires At1g17615. In addition, the up-regulation of *PR1* expression in *exo70B1-3* during *G. cichoracearum* infection was also suppressed by At1g17615 mutation (Fig. 7E). However, activation of MAPK in *exo70B1-3* was not suppressed by At1g17615 mutation (S13D Fig.), indicating that activation of MAPK in *exo70B1-3* is not dependent on At1g17615. Sequence analysis revealed that At1g17615 encodes a TIR-NBS protein, previously designated as *TIR-NBS2* (*TN2*). TN2, and a further 20 genes in *Arabidopsis* ecotype Col-0, lack leucine-rich repeats [38]. Among the nine *tn2* alleles obtained in the screen, three carry missense mutations in the TIR domain, four in the NBS domain, and two carry premature stop mutations (Fig. 7F). TN2 does not express a conserved canonical P-loop, which is likely involved in nucleotide binding, but rather encodes GRS instead of the canonical GKT in the P-loop motif (S14 Fig.). However, the mutant allele *tn-6* (G222R) is in the traditionally defined P-loop (S14 Fig.), and G222 of TN2 is highly conserved among GRS containing NB-TIRs (S14 Fig.), suggesting that the GRS motif of TN2 and its relatives might retain some P-loop function. *TN2* transcripts accumulated at much higher levels in *exo70B1* compared to wild type (Fig. 7G); and the accumulation of *TN2* transcripts was further increased upon *G. cichoracearum* infection (Fig. 7H). The *tn2* mutations suppress *exo70B1*-associated resistance and cell death phenotypes, indicating that enhanced disease resistance and cell death in *exo70B1* requires TN2. The *pad4* mutation suppressed the increased accumulation of *TN2* transcripts in *exo70B1-3* (S15 Fig.), consistent with the observations that *pad4* suppressed *exo70B1-3* associated phenotypes (Fig. 3) and suggesting that the up-regulation of TN2 is important for the *exo70B1-3* mutant phenotype. In addition, *TN2* transcripts over-accumulated in older *exo70B1-3* plants (S16 Fig.), which is consistent with the observations that *exo70B1-3* displays spontaneous cell death after 5 weeks.

To further assess the role of TN2 in plant immunity, we examined the *tn2* single mutant for resistance to fungal and bacterial pathogens. The *tn2* single mutant displayed wild type like responses to virulent fungal pathogen *G. cichoracearum* (S17A-S17C Fig.) and bacterial pathogen *Pto* DC3000 (S17D Fig.), indicating that TN2 does not contribute to basal defense to *G. cichoracearum* or *Pto* DC3000. To further investigate the phenotypes of *exo70B1-3, tn2* and *exo70B1-3 tn2* mutants in response to *Pto* DC3000, we also inoculated those plants by spray infection. As shown in S18 Fig., *exo70B1-3* displayed enhanced resistance to *Pto* DC3000, and the resistance was fully suppressed by the *tn2-1* mutation, which was consistent with our findings usinginfiltration assays. In addition, TN2 was not involved in powdery mildew resistance and mildew-induced cell death in *edr2*, a well characterized powdery mildew resistant mutant, which accumulates higher levels of SA [58] (S19 Fig.).

**Figure 8:**
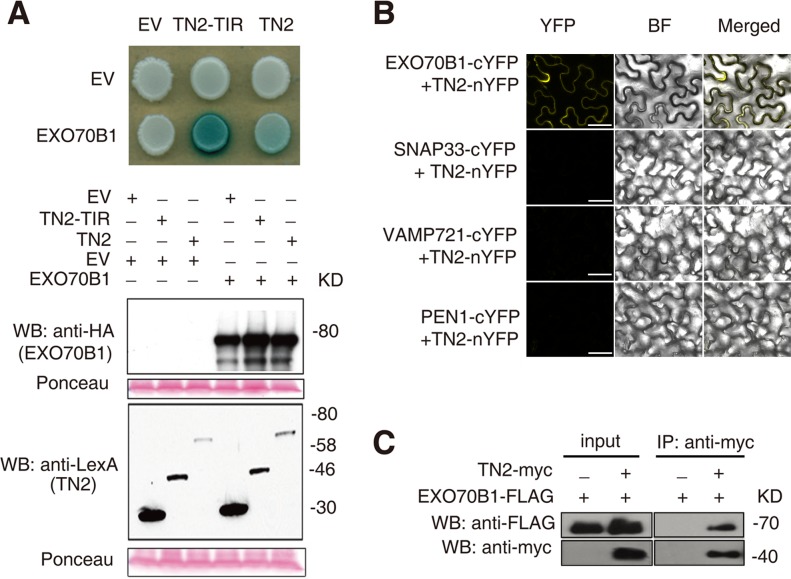
EXO70B1 interacts with TN2. **(A)** EXO70B1 interacted with TN2 in a yeast two-hybrid assay. Yeast cells containing the indicated plasmids were spotted onto the SD-His-Ura-Trp containing X-Gal. EV, empty vector; TN2-TIR, the TIR domain of TN2; TN2, full-length TN2; EXO70B1, full-length EXO70B1. Western blots verify the accumulation of Y2H fusion proteins. Expected sizes of pEG202:EV = 24 kD, pEG202:TN2-TIR = 48 kD, pEG202:TN2 = 67 kD, pJG4-5:EXO70B1 = 84 kD. Ponceau stain of membranes indicates equal loading. **(B)** Interaction between EXO70B1 and TN2 was examined with a BiFC assay in *N. benthamiana*. EXO70B1 and TN2 were fused to the C-terminal or N-terminal fragment of YFP (cYFP or nYFP) respectively. Agrobacterium GV3101 strains carrying different cYFP and nYFP pairs were infiltrated into leaves of *N. benthamiana*. YFP fluorescence was observed only with the transiently expressed EXO70B1-cYFP and TN2-nYFP, but not in the negative controls in *N. benthamiana*. Bar = 50 μm. **(C)** EXO70B1 interacted with TN2 in a Co-IP assay in *N. benthamiana*. *35Spro:EXO70B1-FLAG* was co-expressed with *35Spro:TN2-Myc* in *N. benthamiana* leaves. Total protein was extracted, and subjected to immunoprecipitation of TN2 protein by anti-Myc antibody. Proteins were analyzed by immunoblotting using anti-Myc or anti-FLAG antibody, as indicated. These experiments were repeated three times with similar results.

To further investigate the relationship between TN2 and EXO70B1, we examined whether TN2 interacts with EXO70B1 by yeast two-hybrid assays. As shown in Fig. 8A, EXO70B1 interacts with TN2 and the TIR domain of TN2. We extended this finding by using a BIFC assay in *N. benthamiana* to examine whether EXO70B1 and TN2 associates. *TN2* was fused to the N-terminal fragments of YFP (*YFP^N^*), and was co-transformed to *N. benthamiana* with *EXO70B1-YFP^C^*, *SNAP33-YFP^C^*, *VAMP721-YFP^C^* or *PEN1-YFP^C^*, we observed YFP fluorescence only in leaves co-transformed with the *EXO70B1-YFP^N^* and *TN2-YFP^C^*, but not in leaves co-transformed with *SNAP33-YFP^C^* and *TN2-YFP^N^*, and *VAMP721-YFP^C^* and *TN2-YFP^N^*, or *PEN1-YFP^C^* and *TN2-YFP^N^*, indicating that TN2 can interact with EXO70B1 in *N. benthamiana* (Fig. 8B and S20 Fig.), To further confirm the interaction between EXO70B1 and TN2, we performed Co-IP assays by co-expressing EXO70B1-FLAG and TN2-Myc in *N. benthamiana* leaves. And EXO70B1-FLAG alone was expressed as a negative control. TN2 was immunoprecipitated using anti-Myc antibody, and in the precipitate, EXO70B1 was detected with anti-FLAG antibody only from the leaves that co-expressed both EXO70B1-FLAG and TN2-Myc, not from the negative control leaves (Fig. 8C). Taken together, those observations indicate that EXO70B1 and TN2 interact. These data are consistent with the hypothesis that TN2 or TN2 associated nucleotide binding domain and leucine-rich repeat-containing receptor is a guard directly monitoring the integrity of EXO70B1.

## Discussion

*EXO70B1* belongs to the *EXO70* gene family, and encodes a subunit of the exocyst complex, which plays a key role in anchoring and tethering vesicles to specific sites of the plasma membrane [19]. In yeast and mammals, exocyst complex-mediated vesicle tethering is followed by SNARE protein-mediated fusion to the targeted membrane, an important step in vesicle trafficking that leads to site-directed secretion. We showed that the loss-of-function *exo70B1-3* mutant displayed spontaneous HR-like cell death, enhanced disease resistance to *G. cichoracearum*, and mildew-induced cell death upon infection. Recent work showed that the *exo70B1* mutant displayed HR-like lesions, and accumulated higher levels of SA and *PR1* transcripts [36]. In addition, *exo70B1* exhibited a variety of additional defects, including vacuole anthocyanin accumulation, nitrogen starvation and reduced internalized autophagosomes inside the vacuole, suggesting a role of EXO70B1 in autophagic membrane transport [36]. It will be interesting in the future to address whether these phenotypes also require TN2. Recently, Stegmann et al. reported that the *exo70B1* mutant was more resistant to *H. a.* Emco5, and that *exo70B1* exhibited increased cell death in response to the non-host oomycete pathogen *Phytophtora infestans* [59], consistent with our findings. However, Stegmann et al. also showed that *exo70B1* displayed enhanced susceptibility to *Pto* DC3000 [59], in contrast to our results that *exo70B1* was more resistant to this bacterial pathogen. The differences between our study and Stegmann et al. [59] could be caused by different growth conditions used in these two studies, such as humidity and light intensity.

The HR-like and enhanced disease resistance phenotypes indicate that *exo70B1* behaves like lesion mimic mutant. The *exo70B1-3* associated phenotypes require TN2, a TIR-NBS protein. The fact that we recovered loss of function *tn2* missense changes in a conserved glycine residue in the catalytic domain of the P-loop suggest that activation of TN2 causes the *exo70B1* loss of function phenotypes. Recessive mutations that give rise to enhanced disease resistance and lesion mimic phenotypes are increasingly associated with ectopic activation of NLR immune receptors, as demonstrated for *Arabidopsis acd11* (*accelerated cell death 11*) and *lsd1* (*lesion simulating disease 1*) [52,60,61]. Both *lsd1* and *acd11* result in constitutively activated immune responses in the absence of pathogens; these phenotypes are suppressed by mutations in the TIR-NLR *LAZ5* or the CC-NLRs, *ADR1-L1* and *ADR1-L2*, respectively [52,60,61]. Similar to *exo70B1*, the *lsd1* and *acd11*-associated autoimmune phenotypes correlate with altered levels of SA [52,60,61].

We showed that the truncated NLR protein TN2 associates with EXO70B1, suggesting a direct functional link between TN2 and EXO70B1. Considering the important role of exocytosis in plant immunity, it would not be surprising if some pathogens evolved effectors that target the exocytosis machinery. It is worth noting that the *EXO70* gene family in plants is hugely expanded, with 23 copies in *Arabidopsis*, compared to only one in yeast and animals [55]. Many *exo70* subunits have adopted alternative functions [26], which may contribute to the expansion. One additional reason for this expansion might be that pathogen effectors target EXO70 proteins to manipulate plant immunity. The evolution of new EXO70 family members could allow the plant to evade pathogen effectors and retain antimicrobial secretory function. An alternative evolutionary possibility, and the one most consistent with our data, is that EXO70B1 has retained its function, but evolved associations with TN2, which can be activated by an as yet undiscovered effector that has evolved to manipulate EXO70B1 function. Considering that EXO70B1 contributes to autophagy, another possibility is that the plant may keep the TN2 protein at a low level via degradation of TN2 in an EXO70B1-autophagy dependent pathway. In this scenario, TN2 is activated, and also up-regulated, when EXO70B1 is not functional. Many studies have shown that autophagy plays important role in plant immunity and pathogen triggered cell death. For instance, the *atg5* (*autophagy-related 5*) mutant displays unrestricted chlorotic cell death after infiltration of *Pto DC3000 avrRpm1* [62]. Similarly, the *atg2* mutant shows mildew-induced cell death and enhanced powdery mildew resistance [42]. In addition, Lenz et al. show that *atg5, atg10* and *atg18* mutants display enhanced resistance to *Pto* DC3000 [63]. Hofius et al. show that the *atg7* and *atg9* mutants display enhanced susceptibility to *Pto DC3000* and the Emwa isolate of *H. arabidopsidis*, and cell death triggered by RPS4 and RPP1 was suppressed in those two mutants [64]. Although autophagy is involved in plant immunity, it has not been shown that any NLR receptor protein is specifically degraded by autophagy. It would be very interesting to examine whether the *exo70B1*-related autophagy defects are dependent on TN2, and whether accumulation of TN2 protein is regulated by EXO70B1-associated autophagy.

The TN2 is one of 21 TIR-NBS proteins in the *Arabidopsis* ecotype Col-0 genome [65]. Although the specific roles of TN proteins are not known, some evidence demonstrates that they can contribute to immune system function. For instance, overexpression of some *TN* genes induces cell death in tobacco, and results in stunted growth and high free SA in *Arabidopsis* [39,65]. Li et al. [39,65] showed that cell death in the copine protein double mutant *bon1* (*bonzai1*) *bon3* was partially suppressed by loss-of-function of some NLRs and TN2. However, the suppression was very mild since none of the plants survived to bolt. In addition, *TN2* transcripts did not over-accumulate in *bon1 bon3*. Collectively, these data suggesting that cell death in *bon1 bon3* is different from *exo70B1*.

Some TN proteins can interact with effector proteins from pathogens [39], suggesting that the TN proteins are likely involved in plant immunity. We cannot exclude the possibility that EXO70B1 and TN2 have other cellular functions in addition to biotic interactions. Many *TN* genes occur in complex genomic clusters with TIR-NBS-LRR genes. For instance, *TN2* is in a cluster of 3 TIR-containing genes, including one TIR-NBS-LRR (At1g17600), and another TN gene (At1g17610, *TN1*, also called *CHILLING SENSITIVE 1 or CHS1*). Mutations in *TN1*/*CHS1* caused auto-immunity at low temperature. As expected of a TIR-containing protein, this phenotype required EDS1 and PAD4 [66,67]. However, as no function has been assigned to the TIR-NBS-LRR gene (At1g17600) in this cluster, it will be interesting to examine whether At1g17600 is required for At1g17610 (*TN1*) and At1g17615 (*TN2*) function.

In conclusion, we found that *Arabidopsis* exocyst subunit EXO70B1 associates with the SNARE complex component SNAP33. Loss-of-function of EXO70B1 leads to a syndrome of defense related phenotypes, which require the TIR-NBS2 protein. We speculate that exocyst components could be targets of effectors that are, at least in this case, guarded by TN2. Alternatively, TN2 could function as an adaptor in combination with an undefined classical NLR receptor. However, we note that we did not identify any other NLR receptor in our fairly deep mutant screen (recall that we isolated many alleles of TN2 and PAD4). This would suggest that redundant classical NLR might use TN2 as an adaptor is such a model. Irrespective of the ultimate mechanistic model, we found that EXO70B1 protein interacts with TN2.

Although the role of EXO70B1 in plant immunity is not clear, work by Stegmann et al. showed that the *exo70B1* mutant is compromised in flg22-triggered responses suggesting that EXO70B1 contributes to PTI [35], which makes it a potential target for pathogen effector. Important questions remain to be addressed, including: the nature of the cargos transported by EXO70B1-mediated trafficking, and how those cargos affect plant immunity, as well as how mutation of EXO70B1 activates TN2, a protein that lacks canonical regulatory domains associated with full length NLR receptors, and whether TN2 is involved in the autophagic pathway mediated by EXO70B1. Answering these questions will shed new light on plant immunity.

## Materials and Methods

### Plant materials and growth conditions

The *Arabidopsis thaliana exo70B1-3* mutant was identified from a T-DNA insertion population [14]. T-DNA insertion lines *exo70B1-1* (N328818), *exo70B1-2* (N717829), *exo70B2-1* (SALK_091877C) were from the Arabidopsis Biological Resource Center or Nottingham Arabidopsis Stock Center. The mutant alleles used for the double mutant analyses were as described: *pad4-1* [47], *npr1-1* [16], *eds5-1* [45], *sid2-2* [46], *ein2-1* [68], *coi1-1* [69], *edr1* [70], *edr2* [12], *pmr4-1* [9], *pen1-1* [15]. All of the mutants used were in the Columbia-0 (Col-0) background, except for *coi1-1* (in Col-6 background). Double mutants were created by standard genetic crosses and confirmed by PCR.

*Arabidopsis* plants were grown in a growth room at 22–24°C, at a light intensity of 8000 lux, and a 9-h-light /15-h-dark photoperiod for phenotype analysis or 16-h-light / 8-h-dark photoperiod for seed setting, as described previously [71]. *Nicotiana benthamiana* plants were grown in the same short-day conditions as *Arabidopsis*.

### Fungal infection

Adapted powdery mildew strain *Golovinomyces cichoracearum* UCSC1 [4] was maintained on susceptible *pad4-1* mutant plants. Four-week-old plants were inoculated with powdery mildew using a settling tower at low or high densities [14] [42]. Low spore density was used to quantify the number of conidiophores per colony and high density was used to evaluate hyphae growth and lesion formation. The plants were moved to a growth chamber immediately after infection with *G. cichoracearum* to facilitate growth of the fungi. Hyphae and dead plant cells in the infected leaves were visualized by trypan blue staining [70]. H_2_O_2_ accumulation was detected at 2 dpi by 3,3’-diamino benzidine hydrochloride (DAB) staining [72]. Callose deposition was detected by aniline blue staining [73]. The samples were observed and photographed with an Olympus BX60 microscope. Non-adapted wheat powdery mildew *Blumeria graminis f. sp. tritici* E09 was grown on susceptible Chinese wheat variety Jing411 [74]. Assessment of epidermal cell death in response to *graminis f. sp. tritici* E09 was performed as described [16] with minor modifications. Briefly, four-week-old plants were inoculated with *B. graminis f. sp. tritici* using a settling tower, and the infected leaves was stained with trypan blue, and epidermal single cell death in the infected siytes was visulized with an Olympus BX60 microscope.

*Pseudomonas syringae* infection was performed by leaf infiltration (OD_600_ = 5 ×10^−4^ for growth curve analysis) as described [75], unless otherwise indicated. For spray infection, 5-week-old plants were sprayed with bacterial suspensions (OD_600_ = 0.2) in 10 mM MgCl_2_ containing 0.025% Silwet L-77, and the infected plants were placed under plastic domes for 24 hr.

Inoculation of *Hyaloperonospora arabidopsidis* (*H. a.*) Noco*2* (5 × 10^4^ spores/mL) was performed according to [76].

### Suppressor screen

The *exo70B1-3* seeds were incubated with 0.3% EMS for 16 h. Around 60,000 M2 plants derived from 5,000 M1 plants were scored by infection with *G. cichoracearum*. Plants showing wild type-like phenotypes were selected for further characterization.

### Map-based cloning of *EXO70B1* and *TN2*

The *exo70B1-3* mutant was crossed to ecotype Landsberg *erecta* (L*er*). F2 plants displaying *exo70B1-3* mutant phenotypes were selected for mapping. Using rough mapping markers [77] and our newly developed SSLP, CAPS or dCAPS markers, we narrowed down the mutation to an 83-kb region spanning BAC MCK7 (GenBank accession AB019228) and MQJ2 (GenBank accession AB025632) on chromosome 5. We subsequently designed PCR primers to amplify the coding sequences of these genes. The suppressor gene *TN2* was also identified by standard map-based cloning.

For complementation analysis, a 4.3-kb genomic sequence including 1531 bp upstream of the start codon, and 835 bp downstream of the stop codon of *EXO70B1* was amplified and cloned into binary vector pCAMBIA1300. The construct was then introduced into *Agrobacterium tumefaciens* strain GV3101, and transformed into *exo70B1-3* plants by the floral dip method [78]. Transformants were selected on 1/2 MS medium containing 80 μg/mL hygromycin. T1 transgenic plants were used for phenotyping.

### Quantitative real-time RT-PCR

Extraction of total RNA, synthesis of first-strand cDNA and analysis of gene expression were performed as described previously [79].

### GUS activity assay

The promoter region of *EXO70B1*, including 1531 bp upstream of the start codon, was cloned into the pBI121 vector. Homozygous transgenic plants in the T3 generation were used for GUS activity analysis according to [80].

### SA quantification

SA extraction and HPLC assays were conducted as described previously [49,81].

### Yeast two-hybrid assays

The MATCHMAKER GAL4 Two-Hybrid System 3 (Clontech) was used to examine the interactions between EXO70B1 and other exocyst subunits, as well as PEN1 and SNAP33. *EXO70B1*-pGBKT7 was co-transformed into *Saccharomyces cerevisiae* strain AH109 with SEC3a, SEC5a, SEC6, SEC8, SEC10, SEC15b, EXO84bN (the N terminal domain of EXO84b), PEN1 and SNAP33 fused with pGADT7. The constructs containing SEC3a, SEC5a, or SEC15b fused with pGADT7 were kindly provided by Dr. Viktor Zárský [25]. The constructs containing SEC6, SEC8, SEC10, EXO84bN, PEN1 and SNAP33 fused with pGADT7 were made in this study using primers listed in S1 Table. A single colony grown on SD/-Leu/-Trp plate was incubated in liquid media, the overnight culture was diluted to OD_600_ = 0.5 with sterile water, and a 10 μL drop of dilution was plated on SD/-Ade/-His/-Leu/-Trp plates to test the interaction.

The interaction of EXO70B1 and TN2 was assayed using Gateway-modified versions of the traditional pEG202 and pJG4-5 vectors [82]. pDONR207 Gateway Entry clones containing ORFs (S1 Table for cloning primers) were recombined into pEG202 and pJG4-5 using LR clonase II (Invitrogen). The resulting vectors were sequence verified and then transformed into EGY48 (pEG202) and RFY206 containing the LacZ reporter pSH18-34 (pJG4-5). The resulting strains were mated and diploids selected on synthetic media (SD-HUW). Diploids were spotted onto SD-HUW +X-gal (80 μg/mL). Protein accumulation of fusion proteins in diploids was verified with standard Western blotting using anti-LexA (rabbit pAB, AbCam) and anti-HA (rat mAB, Roche) antibodies.

### BiFC analysis

To create the constructs used in BiFC analysis, the coding sequences of *EXO70B1* and *TN2* were first cloned into pSY736 vector in frame with YFP^N^. The derived YFP^N^-fused-sequences were then amplified and inserted into the Gateway entry vector pDONR207 and subsequently the destination vector pMDC32. Similarly, to create YFP^C^ fused to *EXO70B1, SNAP33, VAMP721* and *PEN1*, the coding sequence of each gene was cloned into pSY735 vector in frame with YFP^C^. The inserts were subcloned into pDONR207 and pMDC32 vectors. *Agrobacterium* GV3101 carrying different YFP^N^ and YFP^C^ pairs were infiltrated into four-week-old *Nicotiana benthamiana* [83]. YFP fluorescence was observed by confocal microscopy (Carl Zeiss LSM710) after 48hr.

### Protein extraction and immunoprecipitation in *N. benthamiana* and *Arabidopsis*

To create the constructs used in coimmunoprecipitation analysis in *N. benthamiana, EXO70B1* was cloned into the pGWB11 vector with C-terminal FLAG fusion using gateway system (Invitrogen). Similarly, *PEN1* was cloned into pEarleyGate 201 vector with 35S promoter and N-terminal HA fusion, while *SNAP33* and *TN2* were cloned into pEarleyGate 203 vector with 35S promoter and N-terminal Myc fusion. Transient expression, total protein extractions and immunoblots were performed as described previously [58]. Briefly, about 20 μg protein was resuspended in sample buffer and separated by SDS-PAGE. The protein was detected by immunoblot with primary antibody anti-GFP (Roche 1:1000) or anti-HA (Sigma 1:1000), and then with anti-mouse (rabbit) HRP-linked secondary antibody, followed by detection with the chemiluminescent HRP substrate kit (Millipore).

For immunoprecipitation, proteins prepared from NB1 buffer [84] were incubated with primary antibody (3 μL /1 mL), at 4 °C for 3 hours with gentle shaking, and 20 μL protein G agarose beads (Millipore) were added and kept shaking for another 3 hours. The beads were washed 4 times with PBS buffer, and resuspended in PBS buffer. The samples were separated by SDS-PAGE and analyzed by immunoblotting. To produce an EXO70B1-GFP fusion, a 3.4-kb genomic sequence, containing 1531bp promoter and 1872 bp coding sequence of *EXO70B1* without the stop codon, was cloned into the binary vector pEGAD [85] between the StuI and AgeI cloning sites to remove the 35S promoter from the vector. The derived *EXO70B1pro:EXO70B1-GFP* construct was introduced into the *A. tumefaciens* strain GV3101 and transformed into *exoB1-3* plants using the floral dip method [78]. To test the interaction between SNAP33 and EXO70B1 in *Arabidopsis*, we amplified *SNAP33* genomic sequence (without stop codon) and fused with an HA tag, and cloned it into pMDC163 vector using gateway system (Invitrogen). And *exo70B1-3* plants expressing *EXO70B1pro:EXO70B1-GFP* were transformed with derived *SNAP33pro:SNAP33-HA* construct, and the T1 plants were used for coimmunoprecipitation immunoblot assays.

To detect MAP kinase activation, 4-week-old plants were inoculated with *G. cichoracearum* for 3 days, and the infected leaves were excised and quickly frozen in liquid nitrogen. Total protein was isolated and subjected to immunoblot with rabbit anti-pTEpY (Cell Signaling), anti-MPK3 or anti-MPK6 (Sigma) as the primary antibody, following the manufacturer’s instructions.

### Oligonucleotide Sequences

The primers used in this work are listed in S1 Table.

## Supporting Information

S1 Fig The *exo70B1-3* mutant displays mildew-induced cell death. Five-week-old uninfected control (upper panel) and plant infected with *G. cichoracearum* at 7 dpi (lower panel) were photographed. Bar = 20 mm.
(PDF)

S2 Fig The *pad4*, but not *sid2, eds5* or *npr1* mutation suppresses spontaneous cell death in *exo70B1-3*. Five-week-old plants grown in the standard short day conditions were photographed. Bar = 20 mm.
(PDF)

S3 Fig The *exo70B1*-mediated resistance and cell death phenotypes did not require *EIN2* and *COI1*. **(A)** Leaves were detached from four-week-old plants and photographed at 7 dpi with *G. cichoracearum*. The *exo70B1 ein2* and *exo70B1 coi1* mutants displayed mildew-induced cell death, similar to the *exo70B1* single mutant.
**(B)** Fungal structures and dead host cells on the surface of leaves at 7 dpi were examined by trypan blue staining. Bar = 100 μm.
**(C)** Fungal growth was assessed in plants by counting the number of conidiophores per colony at 5 dpi. Data represent mean and standard deviation (n > 30). Significant difference is indicated by asterisk (*p* < 0.01; Student’s *t*-test). The experiments were repeated three times with similar results.
(PDF)

S4 Fig The *exo70B1*-mediated resistance and cell death phenotypes were enhanced by *edr1* and *pmr4* mutations. **(A)** Four-week-old plants were inoculated with *G. cichoracearum*. Representative leaves were removed and photographed at 7 dpi.
**(B)** Plant cell death and fungal structures on the infected leaf surface were stained with trypan blue at 7dpi. Bar = 100 μm.
**(C)** Fungal growth was monitored in plants at 5 dpi by counting the number of conidiophores per colony. Lower-case letters indicate statistically significant differences (*p <* 0.01; one-way ANOVA).
(PDF)

S5 Fig Identification and complementation of the *exo70B1-3* mutation. **(A)** The *exo70B1-3* mutation was identified by standard map-based cloning. Markers and BAC clones are indicated. Structure of the *EXO70B1* gene is shown at the bottom. *exo70B1-3* is a T-DNA insertion mutant. The insertion site of *exo70B1-3*, and previous identified T-DNA lines *exo70B1-1, exo70B1-2* are indicated by the triangles.
**(B)-(D)** Complementation of *exo70B1-3* by *Agrobacterium tumefaciens*-mediated transformation. A genomic clone containing *EXO70B1* complemented the *exo70B1-3* mutation. The transcript accumulation of *EXO70B1* was examined by RT-PCR **(B)**. Four-week-old plants were infected with *G. cichoracearum* and photographed at 7 dpi **(C)**. Accumulation of the *PR1* transcripts was examined at 3 dpi by real-time quantitative RT-PCR with *ACT2* as an internal control **(D)**. The asterisk indicates a significant difference from WT (*p <* 0.01; Student’s *t*-test). Bars represent mean and standard deviation from three biological simples. Three independent experiments were performed with similar results.
(PDF)

S6 Fig The *exo70B2* mutant displayed wild type like phenotypes in response to *G. cichoracearum*. **(A)** Leaves of four-week-old plants inoculated with *G. cichoracearum* at 7 dpi.
**(B)** Infected leaves stained with trypan blue to visualize cell death and conidiophores. Bar = 100 μm.
**(C)** Quantification of fungal growth in plants at 5 dpi by counting the number of conidiophores per colony. Statistically significant differences are indicated by lower-case letters (*p <* 0.01; one-way ANOVA).
(PDF)

S7 Fig EXO70B1 interacted with exocyst subunits SEC3a, SEC5a, SEC15b and EXO84bN in yeast two-hybrid assays. **(A)** Yeast two-hybrid assays of interactions between EXO70B1 and other exocyst subunits. Single colonies were resuspended in 150 μL of sterile water, and 10 μL of which was dropped on SD-Ade-His-Leu-Trp plates and incubated at 28°C for 4 days.
**(B)** Overnight culture from single yeast colonies were diluted in sterile water to OD = 0.5, and a serial of dilutions 1:30, 1:900, 1:27000 were prepared, and 10 μL of each dilution was spotted on SD-Ade-His-Leu-Trp plates and incubated at 28°C for 4 days.
(PDF)

S8 Fig *EXO70B1* is expressed in all examined tissues and organs. *EXO70B1* promoter-GUS expression was examined in two-week-old seedling **(A)**, four-week-old plant **(B)**, eight-week-old plant **(C)** and silique **(D)**.
(PDF)

S9 Fig Western blots verify the accumulation of cYFP and nYFP fusion proteins for BIFC assay. Ponceau stain of membranes indicates equal loading.
(PDF)

S10 Fig PEN1 did not interact with EXO70B1 in Co-IP assay. *35Spro:PEN1-HA* and *EXO70B1pro:EXO70B1-GFP* were co-expressed in *N. benthamiana* leaves. Total protein was extracted, and PEN1-HA was immunoprecipitated by anti-HA antibody. Proteins were analyzed by immunoblotting using anti-HA or anti-GFP antibody.
(PDF)

S11 Fig EXO70B1-GFP complemented the *exo70B1* phenotypes. **(A)** Four-week-old plants were infected with *G. cichoracearum* and photographed at 7 dpi.
**(B)** Infected leaves were stained with trypan blue to visualize the fungal structures and dead plant cells. Bar = 50 μm.
(PDF)

S12 Fig Identification of the *tn2* mutations. The *tn2-1* mutation was identified by map-based cloning. Structure of the *TN2* (At1g17615) gene is shown at the bottom.
(PDF)

S13 Fig The *tn2-1* mutation suppressed spontaneous cell death, increased accumulation of H_2_O_2_ and SA levels, but not MAPK activation in *exo70B1* upon powdery mildew infection. **(A)** Plants were grown in the standard short day conditions. Uninfected four-, five- and six-week-old wild type (WT), *exo70B1-3* and *exo70B1-3 tn2-1* plants were photographed. Bar = 20 mm.
**(B)** Infected leaves were stained with DAB to examine accumulation of H_2_O_2_. Bar = 50 μm.
**(C)** Free SA was extracted from leaves of uninfected plants or plants infected with *G. cichoracearum* at 3 dpi.
**(D)** Four-week-old plants were infected with *G. cichoracearum* at 3 dpi. MAPK activation was assessed by the immunoblot analysis using anti-pTEpY antibody, and accumulation of MPK3 or MPK6 protein was examined by immunoblot analysis using anti-MPK3 or anti-MPK6. Ponceau staining is shown as loading control.
(PDF)

S14 Fig Alignment of TN2 (At1g17615) with other *Arabidopsis* TN proteins. The P-loop domain is indicated. TN2 contains a GRS (instead of GKT) motif in the P-loop domain. The mutation of *tn2-6* (G222R), which is in the middle of the P-loop domain, is indicated by an arrow.
(PDF)

S15 Fig Increased accumulation of *TN2* transcripts was suppressed by *pad4*. The relative transcript levels of *TN2* were examined by quantitative real-time PCR and normalized to *ACTIN2*. Bars represent mean and standard deviation from three biological experiments. The asterisk indicates statistically significant difference (*p* < 0.05, Student’s *t*-test).
(PDF)

S16 Fig *TN2* transcripts were up-regulated in older *exo70B1-3* plants. Leaves from plants of different ages were used for RNA isolation. The transcript accumulation of *TN2* was examined by quantitative real-time PCR, with *ACT2* as an internal control. Bars represent mean and standard deviation from three biological experiments. The asterisks indicate statistically significant difference from the wild type (*p* < 0.05, Student’s *t*-test).
(PDF)

S17 Fig *tn2-1* shows wild type-like response to *G. cichoracearum* and *Pto* DC3000. **(A)** Leaves of four-week-old wild type and *tn2-1* plants inoculated with *G. cichoracearum* at 7 dpi. Bar = 10 mm.
**(B)** Infected leaves were stained with trypan blue at 7 dpi. Bar = 100 μm.
**(C)** Fungal growth was assessed in plants at 5 dpi by counting the number of conidiophores per colony.
**(D)** Four-week-old plants of wild type and *tn2-1* were inoculated with suspension of *Pto* DC3000 (OD_600_ = 0.0005) by infiltration. Bacterial growth was monitored at days 0 and 3. Bars represent means and standard deviation of three independent biological replicates.
(PDF)

S18 Fig Phenotypes of *exo70B1-3*, *tn2-1* and *exo70B1-3 tn2-1* in response to *Pto DC3000* by spray infection. Four-week-old plants were inoculated with suspension of *Pto* DC3000 (OD_600_ = 0.2) by spray infection. Bacterial growth was monitored at days 0 and 3. Bars represent means and standard deviation of three independent biological replicates. Statistically significant difference is indicated with the asterisk (*p* < 0.05, Student’s *t*-test).
(PDF)

S19 Fig *tn2-1* did not affect powdery mildew resistance in *edr2*. **(A)** Four-week-old plants were inoculated with *G. cichoracearum*. Leaves were removed and photographed at 7 dpi. Bar = 10 mm
**(B)** Infected leaves were stained with trypan blue to show fungal structures and dead plant cells at 7 dpi. Bar = 100 μm.
**(C)** Fungal growth was monitored in infected leaves at 5 dpi by counting the number of conidiophores per colony. Statistically significant differences were indicated by lower-case letters (*p* < 0.05; one-way ANOVA). Bars represent mean and standard deviation (n>30). The experiments were repeated three times with similar results.
(PDF)

S20 Fig The accumulation of cYFP and nYFP fusion proteins in the BIFC assay was examined by immunoblotting. Ponceau stain of membranes indicates equal loading.
(PDF)

S1 Table List of the primers used in this study. (PDF)
