# Supplementary figures and images for: A Truncated NLR Protein, TIR-NBS2, Is Required for Activated Defense Responses in the exo70B1 Mutant
Source: PLoS Genet. 2015 Jan 24;11(1):e1004945. doi: 10.1371/journal.pgen.1004945 (PMC4305288; doi:10.1371/journal.pgen.1004945)

**Figure S1**

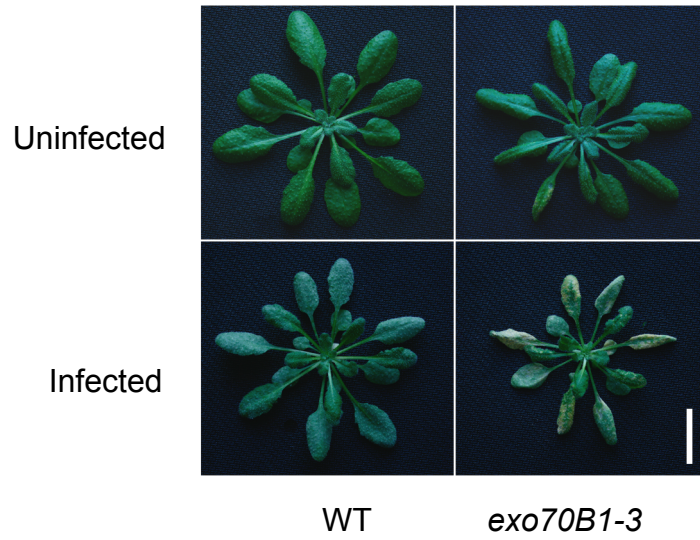

Supplement: S1 Fig — Five-week-old uninfected control (upper panel) and plant infected with G. cichoracearum at 7 dpi (lower panel) were photographed. Bar = 20 mm. (PDF) [file pgen.1004945.s001.pdf]

**Figure S2**

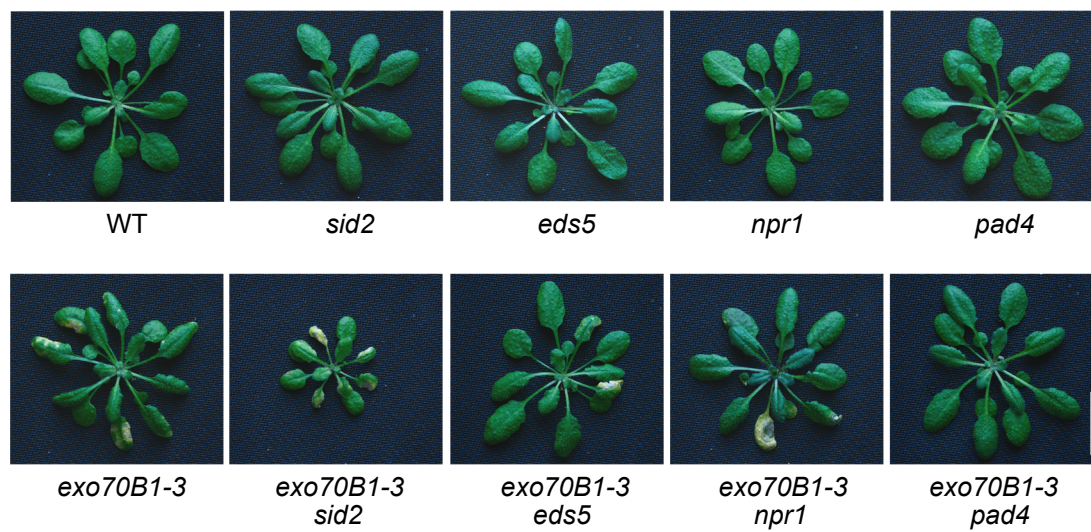

Supplement: S2 Fig — Five-week-old plants grown in the standard short day conditions were photographed. Bar = 20 mm. (PDF) [file pgen.1004945.s002.pdf]

**Figure S3**

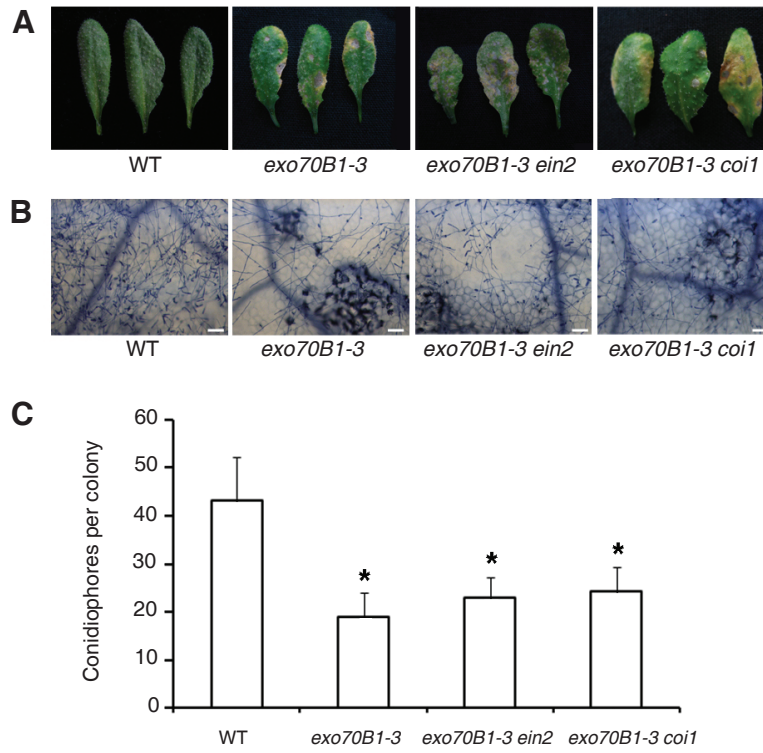

Supplement: S3 Fig — (A) Leaves were detached from four-week-old plants and photographed at 7 dpi with G. cichoracearum. The exo70B1 ein2 and exo70B1 coi1 mutants displayed mildew-induced cell death, similar to the exo70B1 single mutant. (B) Fungal structures and dead host cells on the surface of leaves at 7 dpi were examined by trypan blue staining. Bar = 100 μm. (C) Fungal growth was assessed in plants by counting the number of conidiophores per colony at 5 dpi. Data represent mean and standard deviation (n > 30). Significant difference is indicated by asterisk (p < 0.01; Student’s t-test). The experiments were repeated three times with similar results. (PDF) [file pgen.1004945.s003.pdf]

**Figure S4**

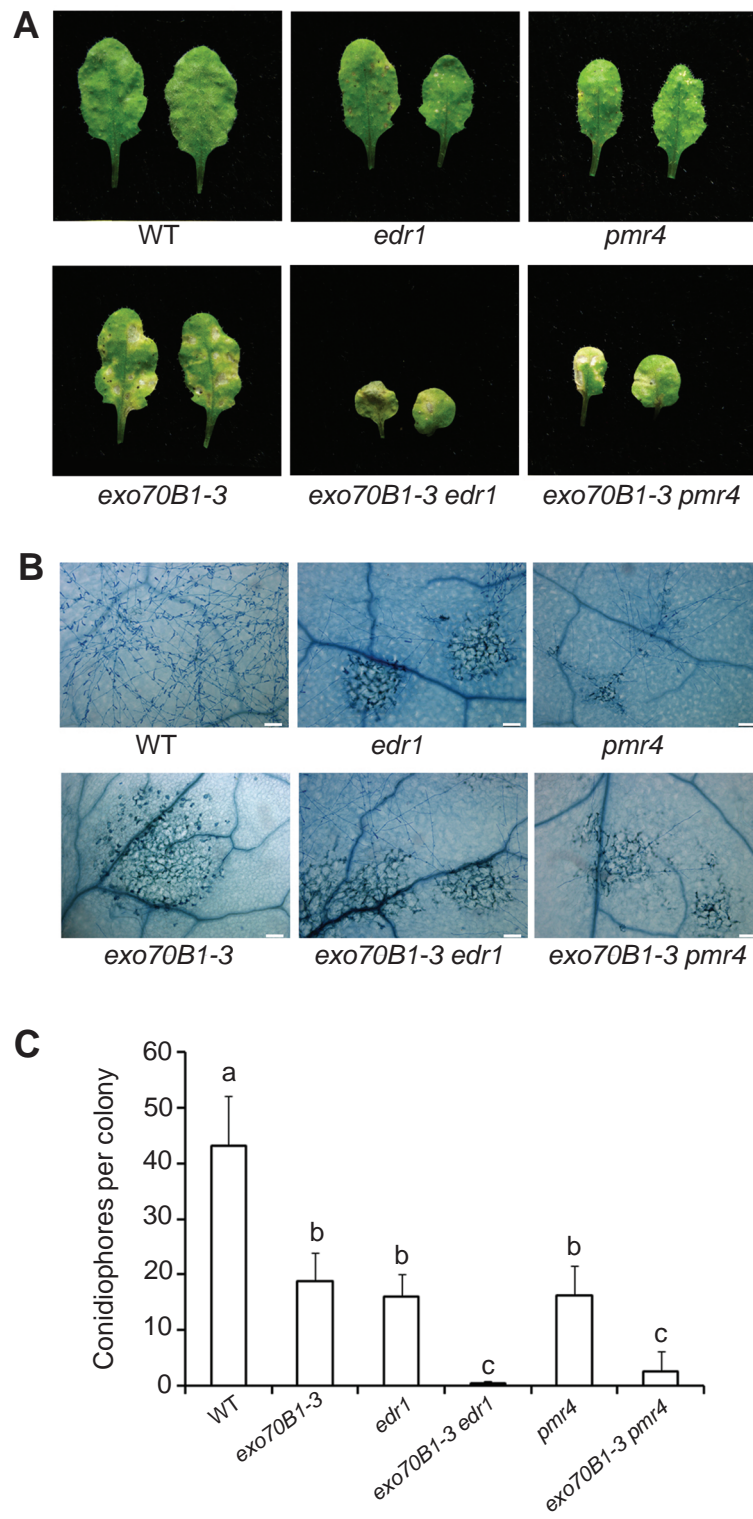

Supplement: S4 Fig — (A) Four-week-old plants were inoculated with G. cichoracearum. Representative leaves were removed and photographed at 7 dpi. (B) Plant cell death and fungal structures on the infected leaf surface were stained with trypan blue at 7dpi. Bar = 100 μm. (C) Fungal growth was monitored in plants at 5 dpi by counting the number of conidiophores per colony. Lower-case letters indicate statistically significant differences (p < 0.01; one-way ANOVA). (PDF) [file pgen.1004945.s004.pdf]

**Figure S5**

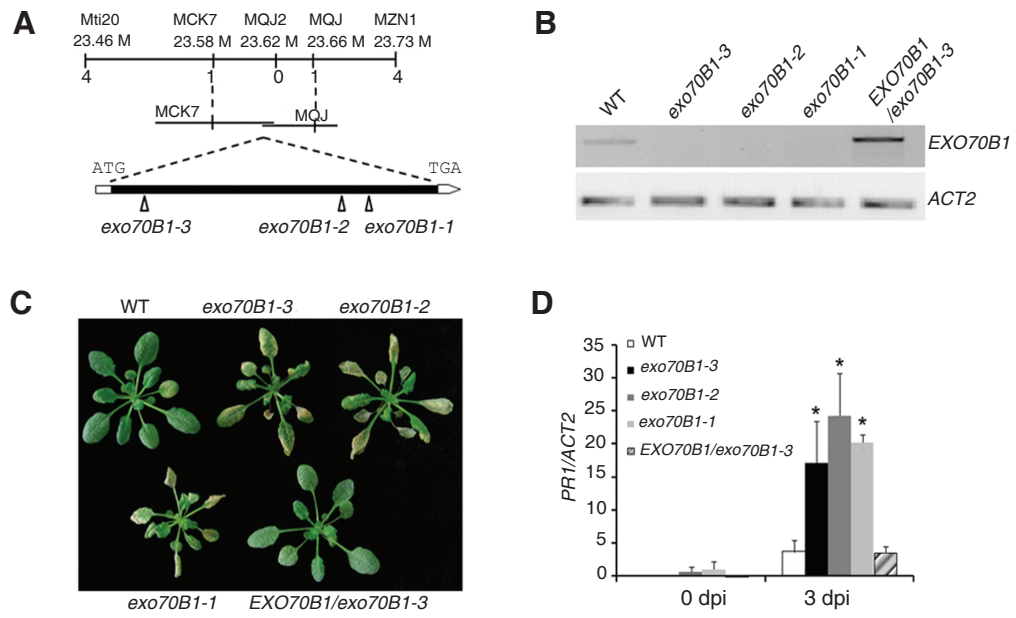

Supplement: S5 Fig — (A) The exo70B1-3 mutation was identified by standard map-based cloning. Markers and BAC clones are indicated. Structure of the EXO70B1 gene is shown at the bottom. exo70B1-3 is a T-DNA insertion mutant. The insertion site of exo70B1-3, and previous identified T-DNA lines exo70B1-1, exo70B1-2 are indicated by the triangles. (B)-(D) Complementation of exo70B1-3 by Agrobacterium tumefaciens-mediated transformation. A genomic clone containing EXO70B1 complemented the exo70B1-3 mutation. The transcript accumulation of EXO70B1 was examined by RT-PCR (B). Four-week-old plants were infected with G. cichoracearum and photographed at 7 dpi (C). Accumulation of the PR1 transcripts was examined at 3 dpi by real-time quantitative RT-PCR with ACT2 as an internal control (D). The asterisk indicates a significant difference from WT (p < 0.01; Student’s t-test). Bars represent mean and standard deviation from three biological simples. Three independent experiments were performed with similar results. (PDF) [file pgen.1004945.s005.pdf]

**Figure S6**

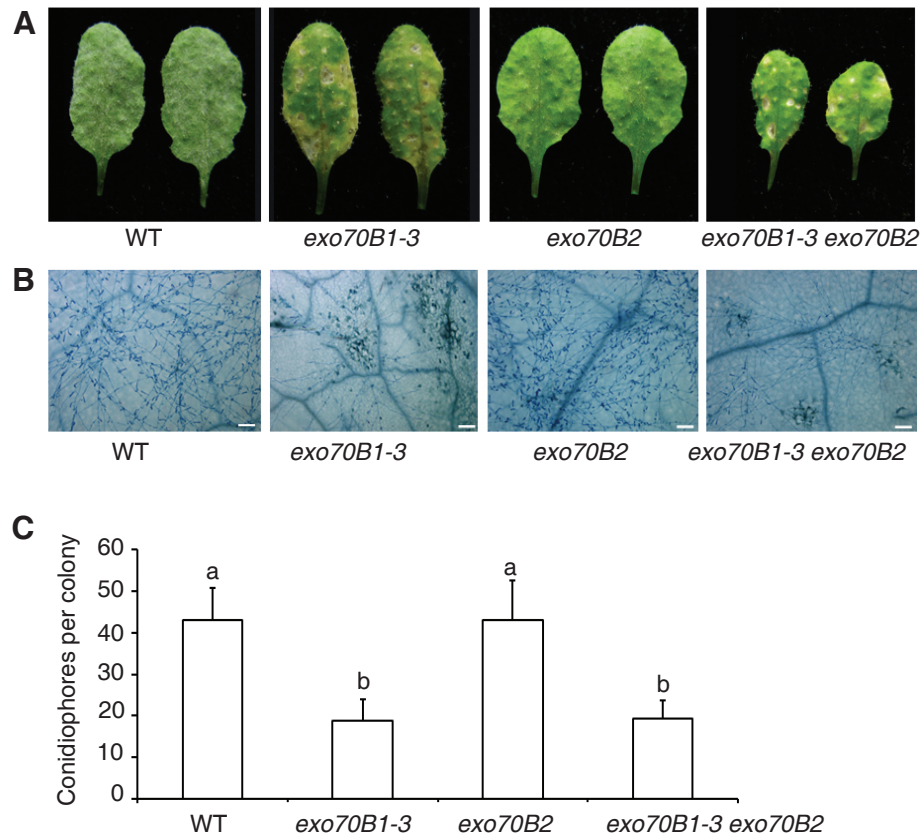

Supplement: S6 Fig — (A) Leaves of four-week-old plants inoculated with G. cichoracearum at 7 dpi. (B) Infected leaves stained with trypan blue to visualize cell death and conidiophores. Bar = 100 μm. (C) Quantification of fungal growth in plants at 5 dpi by counting the number of conidiophores per colony. Statistically significant differences are indicated by lower-case letters (p < 0.01; one-way ANOVA). (PDF) [file pgen.1004945.s006.pdf]

Figure S7

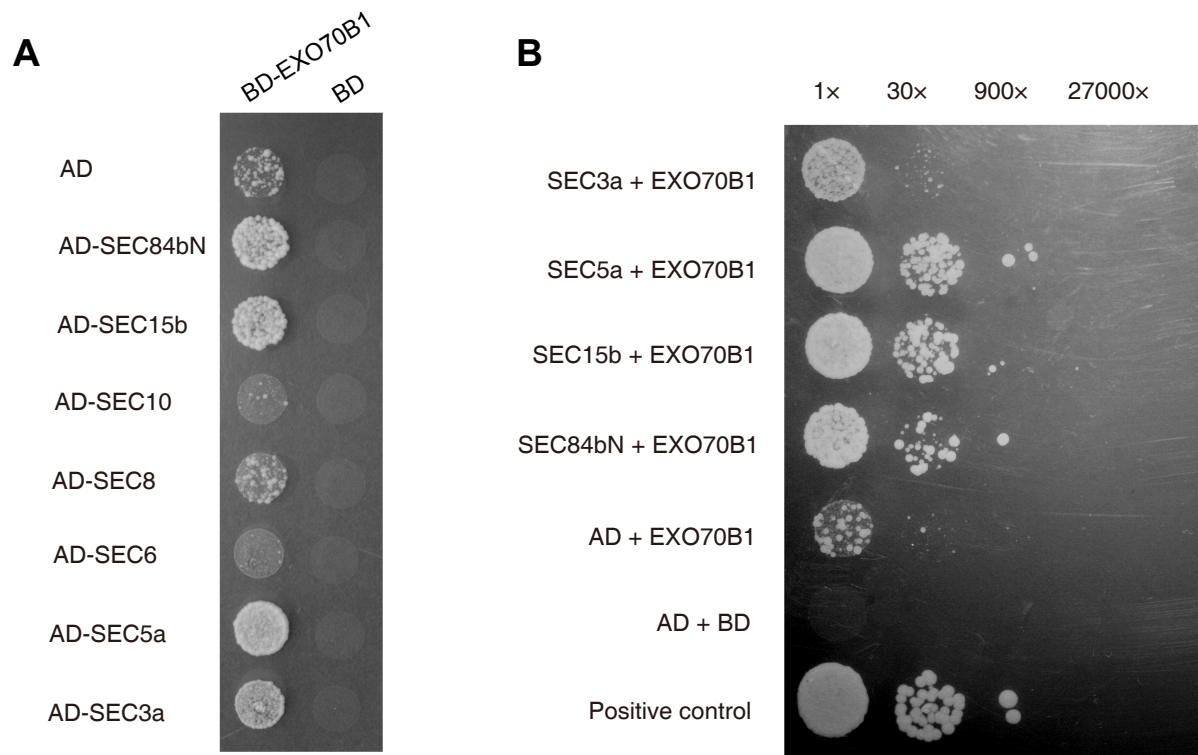

Supplement: S7 Fig — (A) Yeast two-hybrid assays of interactions between EXO70B1 and other exocyst subunits. Single colonies were resuspended in 150 μL of sterile water, and 10 μL of which was dropped on SD-Ade-His-Leu-Trp plates and incubated at 28°C for 4 days. (B) Overnight culture from single yeast colonies were diluted in sterile water to OD = 0.5, and a serial of dilutions 1:30, 1:900, 1:27000 were prepared, and 10 μL of each dilution was spotted on SD-Ade-His-Leu-Trp plates and incubated at 28°C for 4 days. (PDF) [file pgen.1004945.s007.pdf]

**Figure S8**

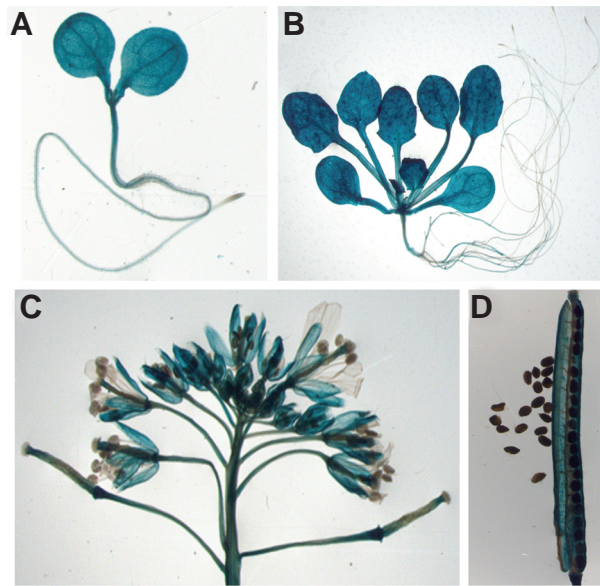

Supplement: S8 Fig — EXO70B1 promoter-GUS expression was examined in two-week-old seedling (A), four-week-old plant (B), eight-week-old plant (C) and silique (D). (PDF) [file pgen.1004945.s008.pdf]

**Figure S9**

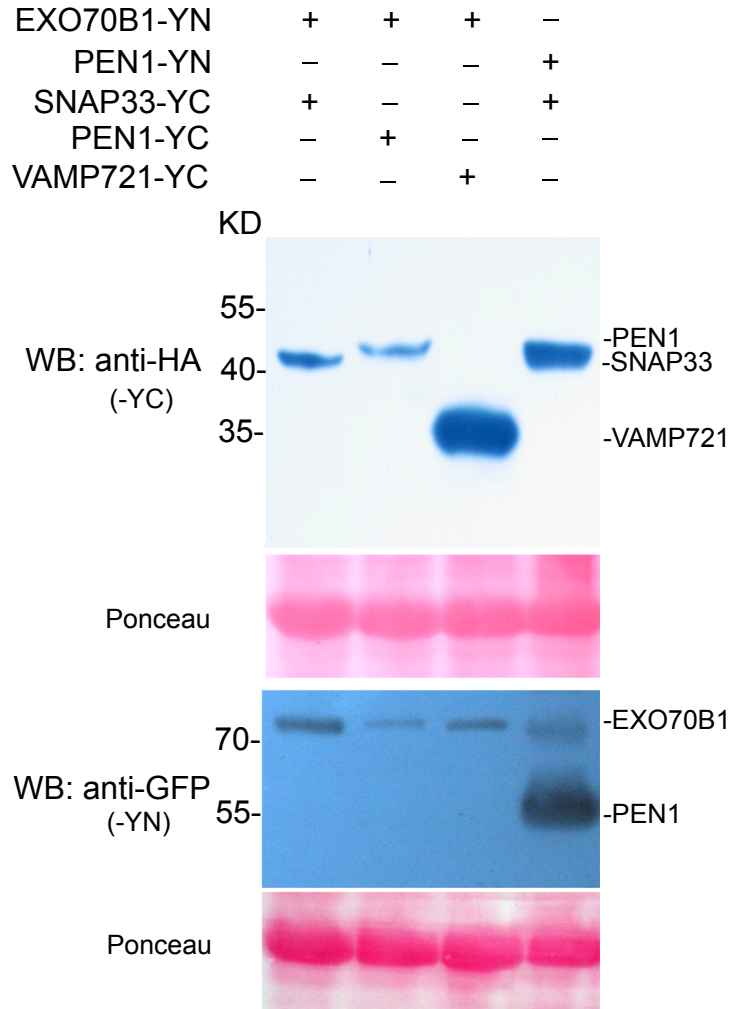

Supplement: S9 Fig — Ponceau stain of membranes indicates equal loading. (PDF) [file pgen.1004945.s009.pdf]

**Figure S10**

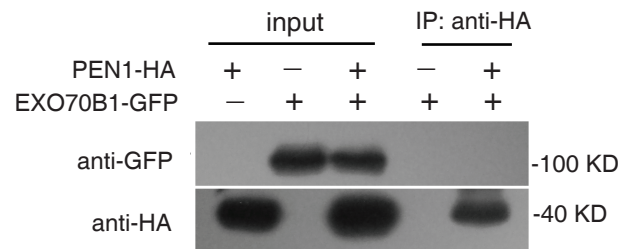

Supplement: S10 Fig — 35Spro:PEN1-HA and EXO70B1pro:EXO70B1-GFP were co-expressed in N. benthamiana leaves. Total protein was extracted, and PEN1-HA was immunoprecipitated by anti-HA antibody. Proteins were analyzed by immunoblotting using anti-HA or anti-GFP antibody. (PDF) [file pgen.1004945.s010.pdf]

Figure S11

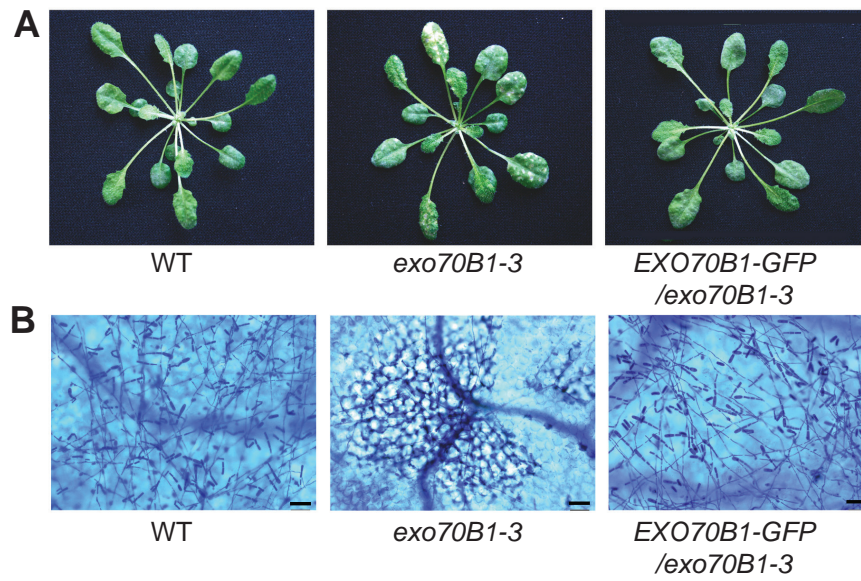

Supplement: S11 Fig — (A) Four-week-old plants were infected with G. cichoracearum and photographed at 7 dpi. (B) Infected leaves were stained with trypan blue to visualize the fungal structures and dead plant cells. Bar = 50 μm. (PDF) [file pgen.1004945.s011.pdf]

**Figure S12**

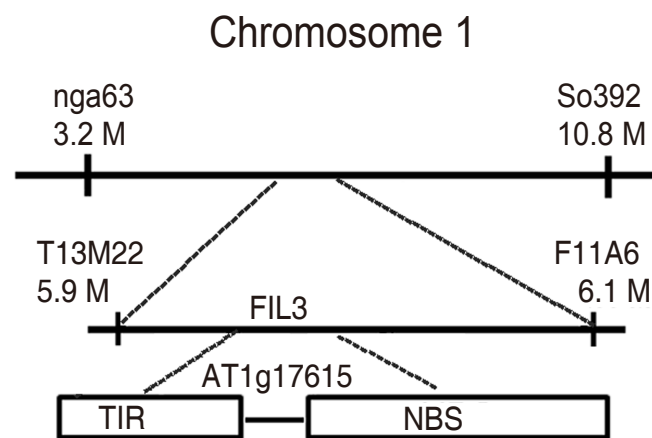

Supplement: S12 Fig — The tn2-1 mutation was identified by map-based cloning. Structure of the TN2 (At1g17615) gene is shown at the bottom. (PDF) [file pgen.1004945.s012.pdf]

**Figure S13**

**A**

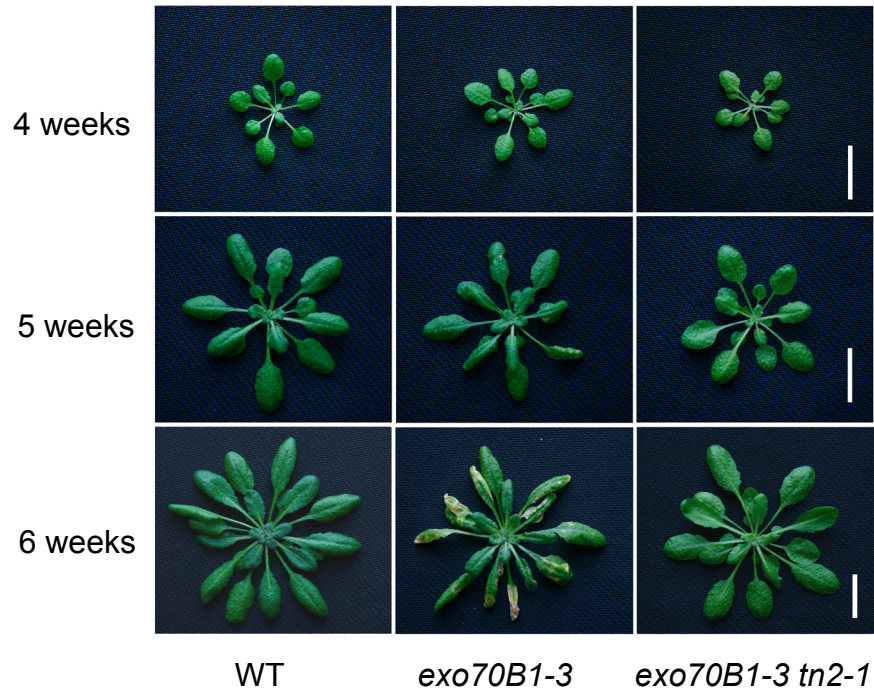

**B**

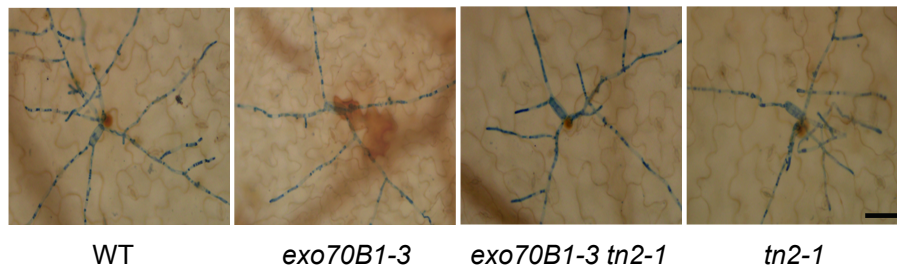

**C**

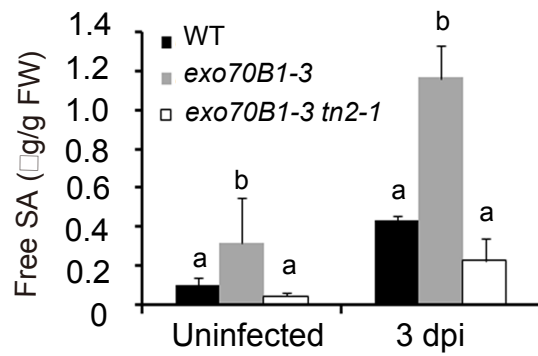

**D**

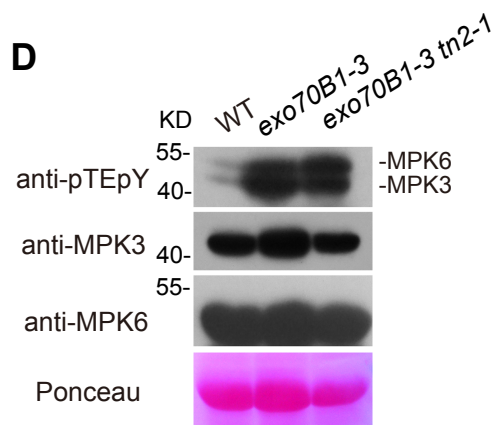

Supplement: S13 Fig — (A) Plants were grown in the standard short day conditions. Uninfected four-, five- and six-week-old wild type (WT), exo70B1-3 and exo70B1-3 tn2-1 plants were photographed. Bar = 20 mm. (B) Infected leaves were stained with DAB to examine accumulation of H2O2. Bar = 50 μm. (C) Free SA was extracted from leaves of uninfected plants or plants infected with G. cichoracearum at 3 dpi. (D) Four-week-old plants were infected with G. cichoracearum at 3 dpi. MAPK activation was assessed by the immunoblot analysis using anti-pTEpY antibody, and accumulation of MPK3 or MPK6 protein was examined by immunoblot analysis using anti-MPK3 or anti-MPK6. Ponceau staining is shown as loading control. (PDF) [file pgen.1004945.s013.pdf]

### Figure S14

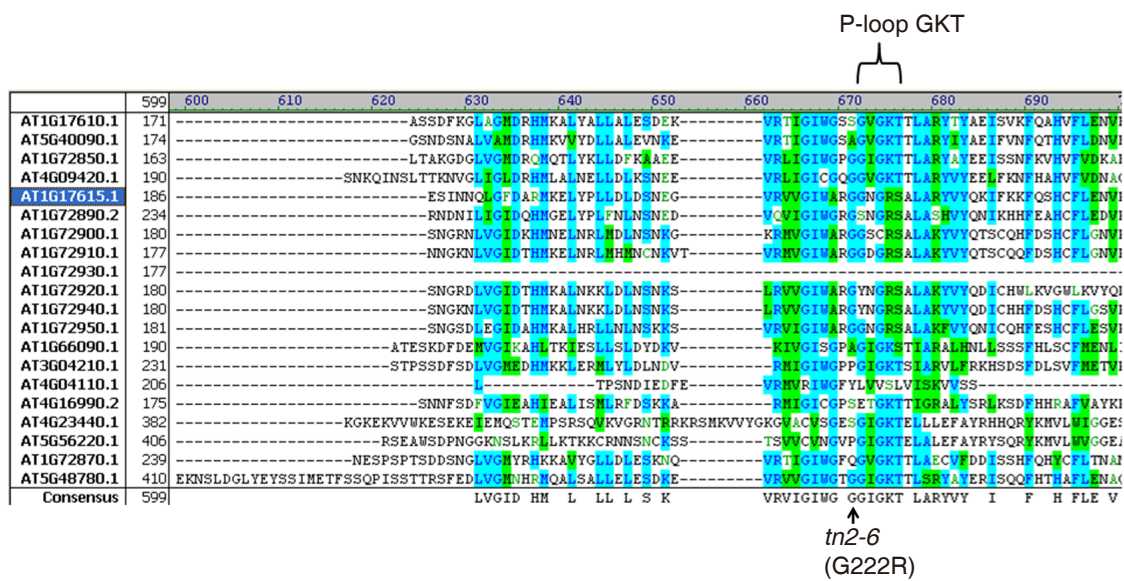

Supplement: S14 Fig — The P-loop domain is indicated. TN2 contains a GRS (instead of GKT) motif in the P-loop domain. The mutation of tn2-6 (G222R), which is in the middle of the P-loop domain, is indicated by an arrow. (PDF) [file pgen.1004945.s014.pdf]

Figure S15

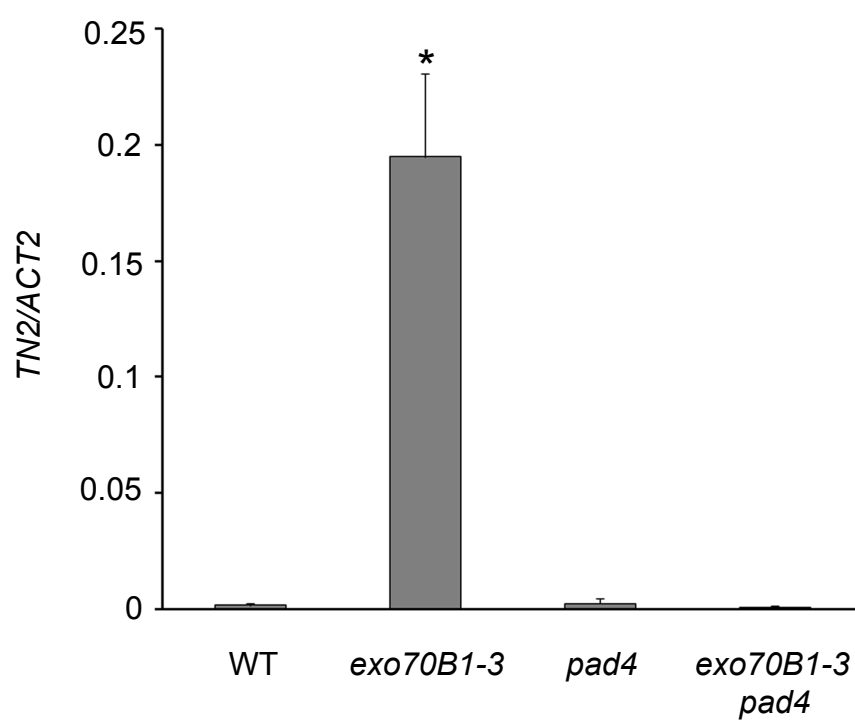

Supplement: S15 Fig — The relative transcript levels of TN2 were examined by quantitative real-time PCR and normalized to ACTIN2. Bars represent mean and standard deviation from three biological experiments. The asterisk indicates statistically significant difference (p < 0.05, Student’s t-test). (PDF) [file pgen.1004945.s015.pdf]

Figure S16

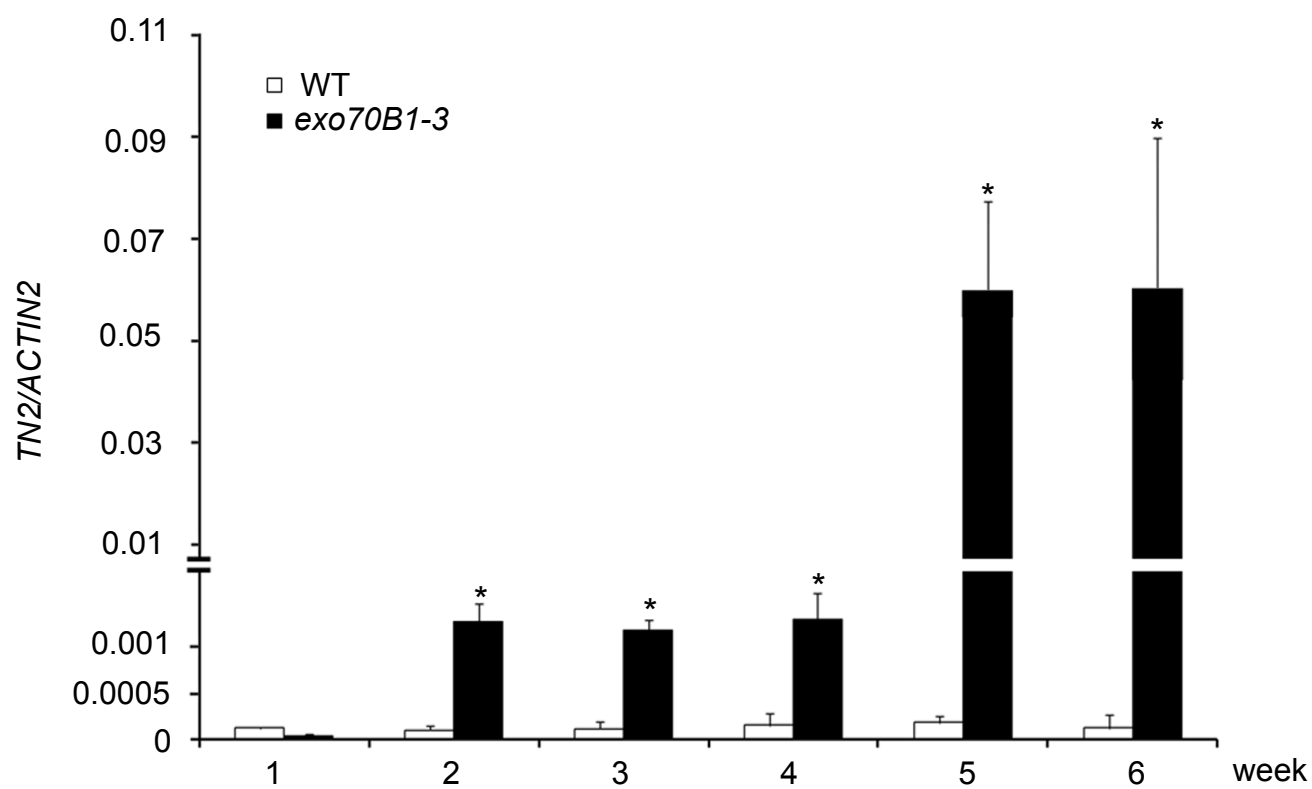

Supplement: S16 Fig — Leaves from plants of different ages were used for RNA isolation. The transcript accumulation of TN2 was examined by quantitative real-time PCR, with ACT2 as an internal control. Bars represent mean and standard deviation from three biological experiments. The asterisks indicate statistically significant difference from the wild type (p < 0.05, Student’s t-test). (PDF) [file pgen.1004945.s016.pdf]

Figure S17

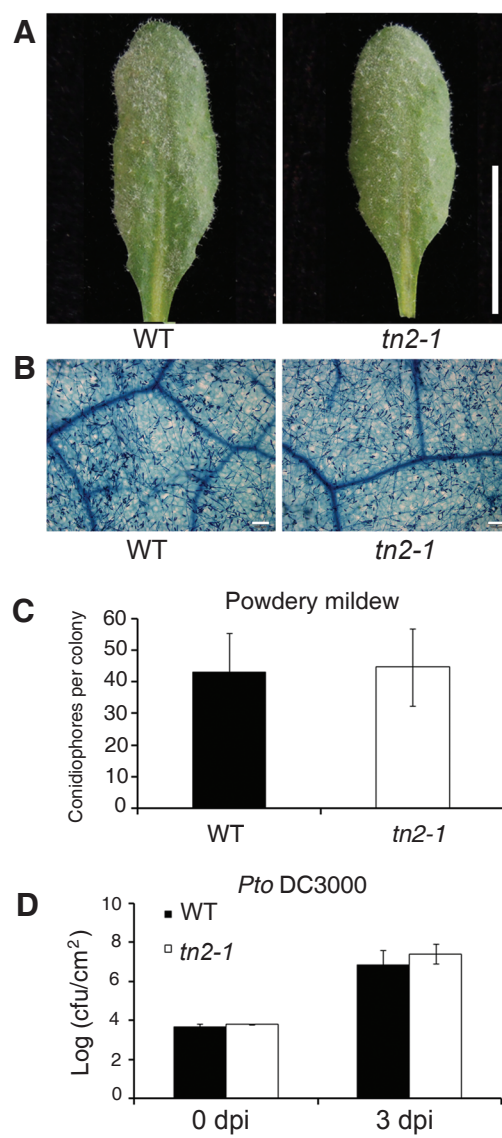

Supplement: S17 Fig — (A) Leaves of four-week-old wild type and tn2-1 plants inoculated with G. cichoracearum at 7 dpi. Bar = 10 mm. (B) Infected leaves were stained with trypan blue at 7 dpi. Bar = 100 μm. (C) Fungal growth was assessed in plants at 5 dpi by counting the number of conidiophores per colony. (D) Four-week-old plants of wild type and tn2-1 were inoculated with suspension of Pto DC3000 (OD600 = 0.0005) by infiltration. Bacterial growth was monitored at days 0 and 3. Bars represent means and standard deviation of three independent biological replicates. (PDF) [file pgen.1004945.s017.pdf]

**Figure S18**

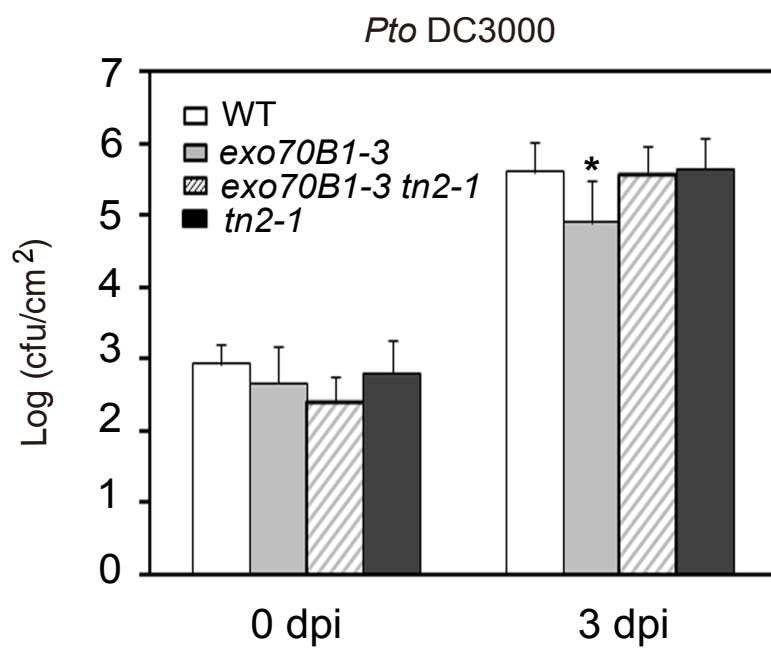

Supplement: S18 Fig — Four-week-old plants were inoculated with suspension of Pto DC3000 (OD600 = 0.2) by spray infection. Bacterial growth was monitored at days 0 and 3. Bars represent means and standard deviation of three independent biological replicates. Statistically significant difference is indicated with the asterisk (p < 0.05, Student’s t-test). (PDF) [file pgen.1004945.s018.pdf]

**Figure S19**

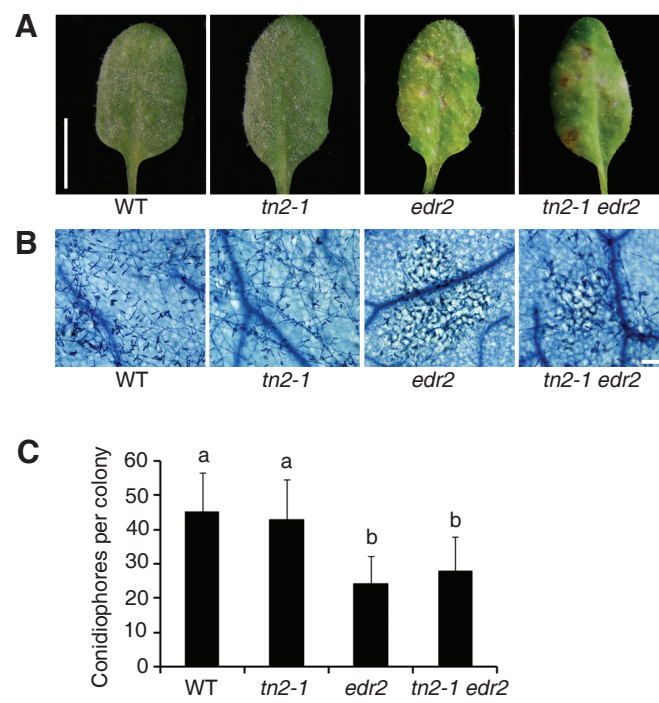

Supplement: S19 Fig — (A) Four-week-old plants were inoculated with G. cichoracearum. Leaves were removed and photographed at 7 dpi. Bar = 10 mm (B) Infected leaves were stained with trypan blue to show fungal structures and dead plant cells at 7 dpi. Bar = 100 μm. (C) Fungal growth was monitored in infected leaves at 5 dpi by counting the number of conidiophores per colony. Statistically significant differences were indicated by lower-case letters (p < 0.05; one-way ANOVA). Bars represent mean and standard deviation (n>30). The experiments were repeated three times with similar results. (PDF) [file pgen.1004945.s019.pdf]

Figure S20

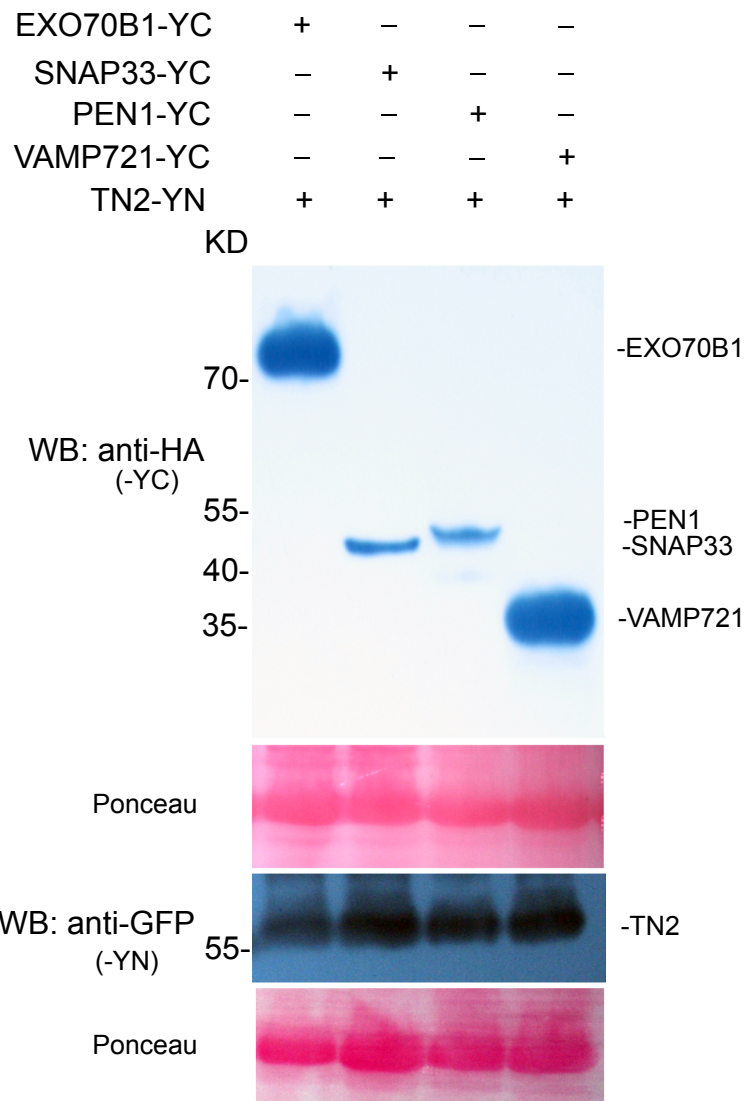

Supplement: S20 Fig — Ponceau stain of membranes indicates equal loading. (PDF) [file pgen.1004945.s020.pdf]
