# Supplementary material for: A Truncated NLR Protein, TIR-NBS2, Is Required for Activated Defense Responses in the exo70B1 Mutant
Source: PLoS Genet. 2015 Jan 24;11(1):e1004945. doi: 10.1371/journal.pgen.1004945 (PMC4305288; doi:10.1371/journal.pgen.1004945)
Supplement: S1 Table — (PDF) [file pgen.1004945.s021.pdf]

Table S1 Primers used in this paper.

**Primer name**	**Purpose**	**Sequence (5´-3´)**
*exo70B1-3*FP	genotyping	CCTGGTAAGTAACAACCAGTTCGT
exo70B1-3 RP	genotyping	AAGATCGTAGTCAGTGAGTGGC
exo70B1-3 LB	genotyping	GGGTGAGATTCCTTGAAGTTGAG
gEx070B1-EcoR IFP	complementation	TGCTGAATTCTAGAGGCTGCATCATCGAAG
gEx070B1-Sal I RP	complementation	CATCGTCGACTCAAGTTTGATGTTGGCAAT
pEx070B1-Hind III FP	promoter-GUS	AAGCTTGCTAGAGGCTGCATCATCGAAGG
pEx070B1-Sma I RP	promoter-GUS	CCCGGGGATTGAAACAGATGTGGAAC
BD-EXO70B1-EcoR IFP	yeast two hybrid	TTCAGAATTCATGGCGGAGAATGGTGAAG
BD-EXO70B1-Sal I RP	yeast two hybrid	GTTGGTCGACTCATTTTCTTCCCGTGGTAG
AD-SEC6-EcoR I FP	yeast two hybrid	CTTGGAATTCATGATGGTCGAAGATCTTGG
AD-SEC6-Xho I RP	yeast two hybrid	GGTTCTCGAGAGTGAGTTTTCGCCACATAG
AD-SEC8-EcoR I FP	yeast two hybrid	AGTCGAATTCATGGGGATTTTCAATGGTTTGC
AD-SEC8-Xho I RP	yeast two hybrid	TTCACTCGAGATGAGAAAGAATTTCCAAAAGGCG
AD-SEC10-Cla I FP	yeast two hybrid	CTGCATCGATCCATGACAGAAGGAATCAGAG
AD-SEC10-BamH I RP	yeast two hybrid	TTATGGATCCTCAGCTCAAGCTTGGCCAC
AD-Exo84bN-EcoR I FP	yeast two hybrid	GGTTGAATTCATGGCGGCGAAGACGGC
AD-Exo84bN-BamH I RP	yeast two hybrid	GGTTGGATCCCTAAACTCCATCTGCTAAGCCATGGATT AGAG
AD-SNAP33-EcoR I FP	yeast two hybrid	CCACGAATTCATGTTTGGTTTAAGGAAATCAC
AD-SNAP33-BamH I RP	yeast two hybrid	TTGTGGATCCCTACTTTCCAAGCAAACG
AD-PEN1- EcoR I FP	yeast two hybrid	GGACGAATTCATGAACGATTTGTTTTCCAGCTC
AD-PEN1-BamH I RP	yeast two hybrid	TTTTGGATCCTCAACGCAATAGACGCCTTG
AD-PEN1- EcoR I（-TM） FP	yeast two hybrid	CCCTGAATTCATGAACGATTTGTTTTCCAG
AD-PEN1-BamH I（-TM） RP	yeast two hybrid	CCTTGGATCCTCAAGTTATGATGATGATGAGAAT
EXO70B1-ORF FP	yeast two hybrid	CAAAAAAGCAGGCTCCATGGCGGAGAATGGTGAA GAGAAGTTAC
EXO70B1-ORF RP	yeast two hybrid	GAAAGCTGGGTGTTTTCTTCCCGTGGTAGTCCCT TTGAAC
TN2-TIR-ORF FP	yeast two hybrid	CAAAAAAGCAGGCTCCACCATGTATTCATCATCGT CTTCTTCTT
TN2-TIR-ORF RP	yeast two hybrid	GAAAGCTGGGTGTCACCAAATTCCAACCACTCTC ACA
TN2-ORF FP	yeast two hybrid	CAAAAAAGCAGGCTCCACCATGTATTCATCATCGT CTTCTTCTTCAG
TN2-ORF RP	yeast two hybrid	GAAAGCTGGGTGTCAAGAAGATTCAGTCCCGGAT ATA
pSY736-YN-Exo70B1-Sal I FP	BIFC	TTCTGTCGACTATGGCGGAGAATGGTGAAGAG
pSY736-YN-Exo70B1-Spe I RP	BIFC	GTCCACTAGTTCATTTTCTTCCCGTGGTAGTC
pSY735-YC-SNAP33-Xho I FP	BIFC	TCTTCTCGAGAATGTTTGGTTTAAGGAAATCAC
pSY735-YC-SNAP33-Spe I RP	BIFC	TCTCACTAGTCTACTTTCCAAGCAAACGGC
pSY735-YC-PEN1-Sal I FP	BIFC	TTTTGTCGACAATGAACGATTTGTTTTCCAG
pSY735-YC-PEN1-Spe I RP	BIFC	TTCCACTAGTTCAACGCAATAGACGCCTTG
pSY735-YC-VAMP721-Sal I FP	BIFC	TTTTGTCGACAATGGCGCAACAATCGTTGAT
pSY735-YC-VAMP721-Spe I RP	BIFC	GGCCACTAGTTTAACACTTAAACCCATGGCAAACT GAG

pSY736-YN-TN2-Sac I FP	BIFC	CCTCGAGCTCATGTATTCATCATCGTCTTC
pSY736-YN-TN2-Spe I RP	BIFC	CCTCACTAGTTCAAGAAGATTCAGTCCCGG
pSY735-YC-EXO70B1- Sal I FP	BIFC	TTCTGTCGACAATGGCGGAGAATGGTGAAGAG
pSY735-YC-EXO70B1-Spe I RP	BIFC	CCTCACTAGTTCATTTTCTTCCCGTGGTAGTCC
attB1-YNFP	BIFC<br>gateway cloning	GGGGACAAGTTTGTACAAAAAAGCAGGCTTCATG GTGAGCAAGGGCGAGGAG
attB2-YN RP	BIFC<br>gateway cloning	GGGGACCACTTTGTACAAGAAAGCTGGGTCCTAT TGGATCCAGATCTGACTAGT
attB1-YC FP	BIFC<br>gateway cloning	GGGGACAAGTTTGTACAAAAAAGCAGGCTTCATG GCCGACAAGCAGAAGAAC
attB2-YCRP	BIFC<br>gateway cloning	GGGGACCACTTTGTACAAGAAAGCTGGGTCCTAG AGGATCCAGATCTGACTAGT
attB1-35S-EXO70B1-Flag FP	Protein interaction in *Nicotianabenthamiana*<br>gateway cloning	GGGGACAAGTTTGTACAAAAAAGCAGGCTTCATG GCGGAGAATGGTGAAGAG
attB2-35S-EXO70B1-FlagRP	Protein interaction in *Nicotianabenthamiana*<br>gateway cloning	GGGGACCACTTTGTACAAGAAAGCTGGGTCTTTT CTTCCCGTGGTAGTCCCTT
attB1-35S-HA-PEN1FP	Protein interaction in *Nicotianabenthamiana*<br>gateway cloning	GGGGACAAGTTTGTACAAAAAAGCAGGCTTCATG AACGATTTGTTTTCCAGCTC
attB2-35S-HA-PEN1RP	Protein interaction in *Nicotianabenthamiana*<br>gateway cloning	GGGGACCACTTTGTACAAGAAAGCTGGGTCTCA ACGCAATAGACGCCTTG
attB1-35S--Myc-SNAP33FP	Protein interaction in *Nicotianabenthamiana*<br>gateway cloning	GGGGACAAGTTTGTACAAAAAAGCAGGCTTCATG TTTGGTTTAAGGAAATCACC
attB2-35S-Myc-SNAP33RP	Protein interaction in *Nicotianabenthamiana*<br>gateway cloning	GGGGACCACTTTGTACAAGAAAGCTGGGTCCTAC TTTCCAAGCAAACGGC
attB1-35S-Myc-TN2 FP	Protein interaction in *Nicotianabenthamiana*<br>gateway cloning	GGGGACAAGTTTGTACAAAAAAGCAGGCTTCATG TATTCATCATCGTCTTCTTCTTC
attB2-35S-Myc-TN2RP	Protein interaction in *Nicotianabenthamiana*<br>gateway cloning	GGGGACCACTTTGTACAAGAAAGCTGGGTCTCAA GAAGATTCAGTCCCGG
gEx070B1GFP-StuI FP	Protein interaction in Arabidopsis	GTTCAGGCCTTAGAGGCTGCATCATCGAAG
gEx070B1GFP-Age I RP	Protein interaction in Arabidopsis	ATGTACCGGTTGTTTTCTTCCCGTGGTAGTCC
NP-SNAP33-HAFP	Protein interaction in *Arabidopsis*	TGACCAAGAAGATTCTTACTATTTGACTCTATTTGA TAGGCCTGTAATGAGCCTAGGTC
NP-SNAP33-HARP	Protein interaction in *Arabidopsis*	CTAAGCGTAATCTGGAACATCGTATGGGTACTTTC CAAGCAAACGGCG
attB1-NP-SNAP33-HAFP	Protein interaction in *Arabidopsis*<br>gateway cloning	GGGGACAAGTTTGTACAAAAAAGCAGGCTTCTGA CCAAGAAGATTCTTACTATT

attB2-NP-SNAP33-HA RP	Protein interaction in *Arabidopsis* gateway cloning	GGGGACCACTTTGTACAAGAAAGCTGGGTCCTAAGCGTAATCTGGAACAT
*ACT2*FP	real-time PCR	TCTCCCGCTATGTATGTCGCC
*ACT2*RP	real-time PCR	GTCACGTCCAGCAAGGTCAAGA
*PR-1*FP	real-time PCR	TTCACAACCAGGCACGAGGAG
PR-1RP	real-time PCR	CTAACCCACATGTTCACGGCG
PR-2 FP	real-time PCR	GAATCAAGGAGCTTAGCCTCACC
*PR-2*RP	real-time PCR	GTAGAGCCGCATTCGCTGGAT
PR-5 FP	real-time PCR	TGTTCATCACAAGCGGCATTG
PR-5 RP	real-time PCR	GGAGTCAATTCAAATCCTCCATCG
PAD4 FP	real-time PCR	CTTTCTTCAGTTAAAGATCAAGGAAGG
*PAD4*RP	real-time PCR	GGCAGAAGTTGTGTGCTAAACG
SID2 FP	real-time PCR	CGCAAGAAGTATGAGTCATGTTCG
SID2 RP	real-time PCR	AACCTGTAACCGAACGACGC
*NPR1*FP	real-time PCR	TAGAGTTGCACTTGCTCAACGTC
*NPR1 R*P	real-time PCR	GTTTCCCGAGTTCCACGGT
ALD1 FP	real-time PCR	CTATGACCCCAAGGACCGATGT
ALD1 RP	real-time PCR	AACCCGCAAACTTAGAGAATGAT
FMO1 FP	real-time PCR	CTCTCTTCTGCGTGCCGTAGTT
*FMO1*RP	real-time PCR	ATCCCTTTATCCGCTTCCTCAA
*TN2*FP	real-time PCR	GGCTCATGAGTCAGAAAG
*TN2*RP	real-time PCR	GAAGATTCAGTCCCGGAT
